# *Streptomyces huangiella* sp. nov., an endophytic actinomycete isolated from *Pheretima aspergillum**,*** a promising candidate for biological pathogen control

**DOI:** 10.1128/spectrum.00717-25

**Published:** 2025-09-17

**Authors:** Dan Huang, Jinli Tan, Jingyang Liao, Liuchong Zhu, Xiang Zhang, Donghua Feng, Wenbin Liu, Xiaobao Jin

**Affiliations:** 1 School of basic medical sciences, Guangdong Pharmaceutical University 71237, Guangzhou, People's Republic of China; 2 Guangdong Provincial Key Laboratory of Pharmaceutical Bioactive Substances, Guangdong Pharmaceutical University 71237, Guangzhou, People's Republic of China; 3 Department of Laboratory Medicine, The First Affiliated Hospital of Guangzhou Medical University, Guangzhou Medical University 26468, Guangzhou, People's Republic of China; Mikrobiologický ústav AV ČR, v. v. i., Třeboň, Czech Republic

**Keywords:** actinobacteria, *Streptomyces huangiella* sp. nov., *Pheretima aspergillum*, phylogenetic, genomics, antimicrobial activity

## Abstract

*Pheretima aspergillum* (E. Perrier) is an annelid of the genus *Pheretima* in the family Megascolecidae, a species of earthworm, whose dried body (Guang Dilong) is a traditional Chinese animal medicine. A new actinobacterium strain, named HD1123-B1^T^, was isolated from the gut contents of *Pheretima aspergillum* caught in the wild in Guangzhou, China. Phylogenetic analysis based on 16S rRNA gene sequences revealed that the strain was primarily identified as a member of the genus *Streptomyces*, sharing more than 98% sequence identity to *Streptomyces endocoffeicus* CA3R110^T^ (98.80%), *Streptomyces coffeae* CA1R205^T^ (98.47%), and *Streptomyces iranensis* HM35^T^ (97.93%). The whole genome size of strain HD1123-B1^T^ was approximately 8.9 Mbp, with 7,464 predicted genes and 71.42 mol% DNA C+G content. Comparative genomic analyses based on digital DNA-DNA hybridization (dDDH) and average nucleotide identity (ANI) values revealed that strain HD1123-B1^T^ represents a novel species within the genus *Streptomyces*. Additionally, 38 biosynthetic gene clusters for secondary metabolites were also predicted in the genome of strain HD1123-B1^T^. Based on LC-MS/MS analysis, the nigericin biosynthesis gene cluster has been completely characterized. The ethyl acetate crude extract of strain HD1123-B1^T^ exhibited remarkable antibacterial activity against gram-positive bacteria (methicillin-resistant *Staphylococcus aureus* ATCC 25213, etc) and gram-negative bacteria *Ralstonia solanacearum* GIM 1.70. Based on these results, HD1123-B1^T^ could be confirmed as an isolate that represents a novel species of the genus *Streptomyces*, for which the name *Streptomyces huangiella* sp. nov. is proposed.

**IMPORTANCE:**

As the largest genus of the phylum Actinomycetes, *Streptomyces* is a kind of microbial resources with great practical and economic value. Due to their unique physiological properties and metabolic capacity, *Streptomyces* have become an important source of bioactive compounds in the world and play an indispensable role in medical and industrial fields. With the advancement of molecular biology and genomics, researchers can more deeply explore the metabolic potential of Actinomycetes, discovering and developing new biologically active compounds. These new compounds may possess various biological activities, such as antibacterial, antiviral, antifungal, and antiparasitic properties, further promoting the development of medicine and related industries. Based on genomic analysis and antibacterial activity, the strain HD1123-B1^T^ was indicated to be a promising candidate for biological pathogen control.

## INTRODUCTION

Among the members of the phylum Actinobacteria, the genus *Streptomyces* is regarded as the richest source of antibiotics (1). In addition to antibacterial compounds, *Streptomyces* species are capable of producing other licensed drugs with antineoplastic, antiviral, anti-parasitic, and immunosuppressive properties, such as doxorubicin (an anticancer drug) (2), rapamycin (an immunosuppressant) (3), and anisomycin (an anti-parasitic agent) (4). Of the natural antibiotics currently known to be derived from microorganisms, over 70% are produced by the genus *Streptomyces* (5). Studies indicate that species of *Streptomyces* can utilize up to 15% of their genome to produce secondary metabolites (6, 7). The genus *Streptomyces* was initially proposed and described by Waksman and Henrici, and which has since become a hot topic in the bioprospecting research for new bioactive natural products (8). As the largest taxon among prokaryotes (9), more than 1,232 species with validly published names of the genus *Streptomyces* have been reported (http://www.bacterio.net, 5 August 2024, date last accessed), making it the most well-described genus in the phylum Actinobacteria. *Streptomyces* species are widely distributed across diverse environments, including soil, water, plants, animals, and extreme habitats (10). The members of the genus are gram-positive, aerobic, high G + C content (69–78 mol%), filamentous actinobacteria that form an extensively branched substrate mycelium and aerial mycelium.

The widespread misuse of antibiotics has led to the emergence of multidrug-resistant pathogens, including *Staphylococcus aureus*, *Enterococcus faecium*, *Escherichia coli*, *Acinetobacter baumannii*, and *Pseudomonas aeruginosa*. This, combined with the diminishing efficacy of existing antibiotics, has made treating infectious diseases increasingly challenging. Consequently, antibiotic resistance has become a critical global public health issue (11–13). During the early stages of antibiotic development, research subjects were long concentrated on soil environmental microorganisms. However, due to the overexploitation of soil microbes, the rediscovery rates of known antibiotics are quite high (14). The metabolites isolated from the same habitat using existing screening models and technologies are largely known compounds, and new breakthroughs are challenging to achieve (15), which has led subsequent research focus to shift toward undeveloped and/or extreme habitats for isolating new actinobacteria, especially new *Streptomyces* species. Like humans, animals have a rich gut microbiome, and the gut bacteria of animals have formed unique and efficient defense systems through evolution to cope with complex natural environments. Apart from the immune function of some active substances secreted by themselves, their symbiotic bacteria can also produce specific metabolites to fend off invading pathogens, thereby protecting their hosts in diverse natural environments. This suggests that gut symbiotic bacteria have the ability to produce a wealth of antibacterial bioactive substances and can be an important source for discovering new antibiotics (16).

The earthworm *Pheretima aspergillum* (*E. Perrier*) is a type of annelid belonging to the family Megascolecidae, whose dried body is commonly known as "Guang Dilong" (17). It is mainly distributed in Guangxi and Guangdong and is a traditional Chinese medicine in the Lingnan region, known for its effects of antipyretic, anti-asthmatic, and diuretic and calms the mind (18). *Pheretima aspergillum* feeds on soil organic matter, and its gut microbiota can efficiently decompose stubborn organic substances such as cellulose and lignin, as well as degrade pollutants like pesticides and microplastics. *Pheretima aspergillum* survives in low-oxygen, high-pathogen-risk soil environments, and its gut microorganisms may assist the host in resisting pathogens or enhancing metabolic adaptability. Some of its pharmacological activities, such as anti-thrombotic and anti-inflammatory effects, may also be related to the metabolic products of its symbiotic microorganisms (19, 20). Research has found that the gut of *Pheretima aspergillum* contains a large number of bacteria, including Proteobacteria, Actinobacteria, Firmicutes, Verrucomicrobia, Chloroflexi, Planctomycetes, Acidobacteria, and Verrucomicrobia, with Actinobacteria being the most abundant at the phylum level (with an average relative abundance of 28.96%). Some strains are capable of producing secondary metabolites that have active enzyme properties (21). Currently, there is limited research on the antibacterial activity of secondary metabolites from actinobacteria in the gut of earthworms, with most studies focusing directly on the antibacterial effects of earthworm castings. I. U. Nwankwo et al. isolated three strains of *Streptomyces* from earthworm castings, which exhibited antibacterial activity against *Staphylococcus aureus*, *Klebsiella pneumoniae*, and *Salmonella* spp., with MIC values of 6.25, 6.25, and 1.56 mg/mL, respectively (22). Additionally, Balachandar R et al. isolated five strains of Actinobacteria from the gut of earthworm, three of which showed significant inhibitory effects against *Staphylococcus aureus*, *Bacillus circulans*, *Bacillus subtilis*, and *Escherichia coli* (23). In our preliminary study, a total of 414 gut symbiotic strains were isolated from *Pheretima aspergillum* gut samples using traditional culture methods, containing 3 phyla, 11 orders, 16 families, and 19 genera. Notably, Actinobacteria predominated among these isolates, comprising 332 strains, with *Streptomyces* representing a remarkable 97.3% of all isolated symbiotic actinobacteria. Comparative analysis of 16S rRNA sequences revealed that 23 *Streptomyces* strains may represent potential novel species. As a kind of microbial resource from a special environment, the screening of actinobacteria from the intestinal contents of *Pheretima aspergillum* is also an important pathway to discover new species and bioactive secondary metabolites.

In the study of actinobacterial resources in the intestinal tract of animals, a strain of *Streptomyces* HD1123-B1^T^ with significant antibacterial activity, was isolated from the gut contents of *Pheretima aspergillum* captured in the wild in Guangdong Province. This study identified HD1123-B1^T^ as a new species of the genus *Streptomyces,* and its name *Streptomyces huangiella* sp. nov. is proposed (=GDMCC 4.423^T^ =JCM 37538^T^). The whole genome of the strain was sequenced and analyzed, and the clusters of secondary metabolite biosynthesis genes were studied to guide the discovery of new natural products. Genomic analysis revealed that the strain possesses various biosynthetic potentials, with one gene cluster showing 100% similarity to the biosynthetic gene cluster of Nigericin, which may be related to its antibacterial activity. Based on the antibacterial activity and biosynthetic potential results, strain HD1123-B1^T^ could be an important source of novel antibacterial metabolites.

Nigericin is a polyether ionophore antibiotic produced by *Streptomyces*, with the molecular formula C_40_H_68_O_11_. It possesses a unique ability to transport ions across membranes, selectively binding to K^+^ and H^+^ ions (24). By disrupting the membrane potential and pH homeostasis, it exerts its antibacterial effects. This compound was initially isolated from *Streptomyces hygroscopicus*, and its structural features include a polyether backbone and a terminal carboxylic acid group, which enable it to form stable cation complexes (25). Studies have shown that nigericin has the ability to regulate the NLRP3 inflammasome, demonstrating potential applications in cancer immunotherapy and the intervention of inflammatory diseases (26). In addition to its anticancer activity, nigericin exhibits significant inhibitory effects against gram-positive bacteria such as methicillin-resistant *Staphylococcus aureus* (MRSA), vancomycin-resistant Enterococcus (VRE), and penicillin-resistant *Streptococcus pneumoniae*, with a minimum inhibitory concentration (MIC) range of 0.004–0.125 μg/mL, which is lower than that of commonly used clinical antibiotics (27). Notably, this study found that the strain *Streptomyces huangiella* sp. nov. HD1123-B1^T^, derived from the gut of the earthworm, carries a complete nigericin biosynthetic gene cluster, with 100% similarity to known gene clusters. This ionophore antibiotic has previously only been found in terrestrial *streptomycetes*, and the unique evolutionary position of this strain as a symbiont in the gut of *Pheretima aspergillum* may confer new structural characteristics and biological activities to its secondary metabolites.

## MATERIALS AND METHODS

### Isolation and culture conditions

Strain HD1123-B1^T^ was isolated from the gut contents of *Pheretima aspergillum* captured in the wild in the medicinal plant garden of Guangdong Pharmaceutical University in Guangzhou, China in the summer of 2023 (latitude：N 23.055278°, longitude：E 113.408818°). This strain was obtained from a mixed sample of gut contents of five *Pheretima aspergillum* captured from different collection soil and preserved as a suspension of mycelia in glycerol (30% vol/vol) at −80°C. The surface disinfection method was used to the samples of *Pheretima aspergillum* captured from the wild in Guangzhou (28): they were starved for 48 h and fed with water only. The samples were rinsed with running water, soaked in 75% ethanol for 2 minutes, disinfected with 3.5% sodium hypochlorite for 2 minutes, then soaked in 75% ethanol for 1 minute, and finally washed with sterile water three times before dissection under sterile conditions. The intestinal contents were removed and placed in a sterile mortar for full grinding, and the grinding solution was diluted to concentrations of 10^−1^, 10^−2^, and 10^−3^ with sterile water. Subsequently, a 200 µL aliquot was evenly distributed on separation culture media (Gause’s synthetic agar no.1 medium, a starch-based medium selective for actinomycetes, GD Huankai MST) containing potassium chromate (75 mg/mL) and ampicillin (100 mg/mL) (29). After 14 days of incubation at 28°C, single colonies were selected for purification on International *Streptomyces* Project (ISP) medium no. 2 (ISP 2 medium) (30). The purified single colonies were preserved in glycerol (30% vol/vol) at −20°C and −80°C.

### Morphology 

Strain HD1123-B1^T^ was inoculated onto tryptone-yeast extract, yeast extract-malt extract, oatmeal, inorganic-salts starch, glucose-asparagine, and peptone-yeast extract-iron (International *Streptomyces* Project media, ISP 1-6) (30), Czapek–Dox Medium (CA, GD Huankai MST) (31), nutrient agar (NA, GD Huankai MST), Luria-Bertani medium (LB, GD Huankai MST), and tryptose soya agar (TSA, GD Huankai MST) and incubated at 28°C for 14 days. Cultural characteristics, including growth conditions, colony morphology and color; the presence of substrate mycelium and aerial mycelium; and the production of pigment were observed, with the color of aerial and substrate mycelium and soluble pigments determined using the ISCC-NBS color charts. Gram staining was carried out by using the standard Gram stain described by Smibert and Krieg (32). The production of melanin was observed on ISP 6 medium following the method by Sandoval-Powers M et al. (33). Strain HD1123-B1^T^ was cultured on ISP 2 liquid medium at 28°C for 7 days, and the morphology of mycelia and spores was observed by scanning electron microscopy (SEM). The biomass was collected for fixation, gradient dehydration, and drying. The dried samples were coated with a thin layer of gold in a vacuum and imaged using SEM (Hitac Reguius 810000).

### Physiology and biochemical properties

The effects of different temperatures, pH, and NaCl range on the growth of strains cultured on ISP 2 media were determined according to the standard methods described by Shirling and Gottlieb (30). The strain was inoculated into ISP 2 agar and incubated at various temperatures (4, 10, 20, 28, 35, 40, and 45°C) for 14 days to observe growth conditions and temperature tolerance. The NaCl tolerance was examined on supplemented ISP 2 agar with adding 0%–13% (wt/vol) NaCl (at intervals of 1% wt/vol) and incubating at 28°C for 14 days. The pH range for growth was determined in ISP 2 agar with pH 5.0–12.0 (at intervals of 0.5 pH unit) at 28°C for 7 days. The utilization of various carbon sources was determined using HKM Bacterial Biochemical Identification Tube based on the methods described by Pridham TG and Gottlieb D (34). Other physiological and biochemical tests, including catalase, oxidase, starch hydrolysis, nitrate reduction, urease activity (35), hippurate hydrolysis, citrate utilization, tryptophan degradation, hydrogen sulfide production, melanin production, lipase hydrolysis (Tween 20, 40, 60, and 80 at 1% vol/vol), aescin hydrolysis, gelatin liquefaction, casein hydrolysis (36), and MR-VP test, were examined according to the methods described by Shirling EB and Gottlieb D (30), Smibert, R., Krieg, N., et al. (37), Pridham TG and Gottlieb D (34), Maiti PK and Mandal S (38), and Cowan et al. (39).

### DNA extraction and 16S rRNA gene analysis

Genomic DNA was extracted from the strain HD1123-B1^T^ according to the method of the Sangon Biotech Ezup Column Bacteria Genomic DNA Purification Kit. PCR amplification of the 16S rRNA gene sequence was carried out using the forward primer 27F (5′-AGAGTTTGATCCTGGCTCA-3′) and the reverse primer 1492R (5′-GGTTACCTTGTTACGACTT-3′) (40). The obtained PCR purification products were cloned into the pMD19 T Vector (TaKaRa) and then transformed into *Escherichia coli* DH5α. The recombinant clones were sent to Beijing GenomicsInstitution (BGI) (https://www.bgi.com/) for sequencing. The almost complete 16S rRNA gene sequence of strain HD1123-B1^T^ was obtained and compared with wild-type strains available in the NCBI database (https://blast.ncbi.nlm.nih.gov/Blast.cgi) and the EzBioCloud database (https://www.ezbiocloud.net/identify) to identify closely related strains. The 16S rRNA gene sequence of the strain was retrieved using NCBI BLAST (National Center for Biotechnology Information, Basic Local Alignment Search Tool; https://blast.ncbi.nlm.nih.gov/Blast.cgi;) and then submitted to the GenBank database to obtain the login number. Multiple sequence alignments were performed using the Clustal W algorithm in Molecular Evolutionary Genetics Analysis (MEGA) version 11.0 (41), trimmed manually where necessary. The evolutionary distances were calculated based on the Kimura two-parameter model (42). Phylogenetic trees were constructed using the neighbor-joining method (43), maximum-parsimony method (44), and maximum-likelihood method (45). The stability of the phylogenetic tree topology was assessed using the bootstrap method with 1,000 repetitions (46).

Three of the most closely related phylogenetic relatives to strain HD1123-B1^T^ based on the 16S rRNA gene sequence were selected for comparative analysis. These reference strains included *Streptomyces coffeae* CA1R205^T^, *Streptomyces iranensis* HM35^T^, and *Streptomyces endocoffeicus* CA3R110^T^.

### Genome sequencing and analysis

The strain HD1123-B1^T^ was cultured in ISP 2 liquid medium at 28°C for 7 days to produce the biomass required for genomic sequencing. The cells were collected by centrifugation and sent to Beijing Qinke Biotechnology Co., Ltd. for sequencing. Whole-genome sequencing was performed on the Nanopore sequencing platform following the standard protocol provided by Oxford Nanopore Technologies (ONT), including DNA extraction from the cells, sequencing, and post-sequencing data processing. To construct the sequencing library, genomic DNA was fragmented, size-selected, A-tailed, ligated to paired-end adapters, and PCR-amplified, yielding 350 bp inserts. The raw data format generated by Nanopore sequencing is in binary fast5 format, containing all original sequencing signals, with each read corresponding to a single fast5 file. After base calling using the Guppy v3.2.6 software from the MinKNOW software package, the fast5 format data were converted to fastq format for subsequent analysis. Then, low-quality and short reads (length <2,000 bp) were filtered out to obtain the final data set. The filtered reads were assembled using Canu v1.5 (47). The assembly results were corrected using third-generation reads with Racon v3.4.3 software, and circularization and adjustment of the starting point were performed using Circlator v1.5.5 software. Further error correction was carried out using second-generation data with Pilon v1.22 software to achieve a more accurate genome for subsequent analysis. Gene composition prediction was performed using Prodigal v2.6.3 (48). Using GenBlastA v1.0.4, functional genes were masked while scanning the entire genome. Premature mutations and frameshift mutations were analyzed using GeneWise v2.2.0 on assumed candidates. Transfer RNA (tRNA) genes were predicted using tRNAscan-SE v2.0 (49), and ribosomal RNA (rRNA) genes were predicted using Infernal v1.1.3 (50). Repetitive sequences were predicted using RepeatMasker (51). Genomic islands in bacterial genomes were predicted using IslandPath-DIMOB v0.2 (52). Prophages in bacterial genomes were predicted using PhiSpy v2.3 (53). Secondary metabolic gene clusters were predicted using antiSMASH v8.0 (54), and promoter prediction was conducted using PromPredict v1 (55). Finally, based on the assembled and predicted genomic information, such as tRNA, rRNA, repetitive sequences, GC content, and gene function information, the genomic circle map of strain HD1123-B1^T^ was created using Circos v0.66 (56). To further analyze the genomic sequence assembled from the new strain, the RAST server version 2.0 was used for rapid annotation (57). For functional annotation, the predicted proteins were subjected to BLAST (e-value: 1e^−5^) against Kyoto Encyclopedia of Genes and Genomes (KEGG) (kegg_201703), eggNOG (v4.0), and Blast2GO V2.5 (58) for gene ontology (GO) annotation (release version 20180910). The whole 16S rRNA gene sequence and whole-genome sequence (WGS) of strain HD1123-B1^T^ submitted to the GenBank/EMBL/DDBJ database, have been deposited at NCBI Genbank under accession numbers PP935229 and CP159919, respectively.

### Whole-genome comparison and systematic genome analysis

To determine the genomic relatedness between strain HD1123-B1^T^ and its phylogenetic relatives with available genome sequences, the average nucleotide identity (ANI) was calculated using the OrthoANI software package (59). The digital DNA-DNA hybridization (dDDH) values were calculated using the recommended settings of the Genome-to-Genome Distance Calculator (GGDC) version 2.1 (60). To obtain a more comprehensive phylogenomic analysis to determine the uniqueness of the strain, the genomic sequence of strain HD1123-B1^T^ was uploaded to the Type Strain Genome Server (https://tygs.dsmz.de) (61). The strain was compared with closely related type strain genomes available in the TYGS database via the MASH algorithm (62) and the Genome BLAST Distance Phylogeny (GBDP) method (63). A balanced minimum evolution tree with branch support was constructed using intergenomic distances via FASTME 2.1.6.1 (64). Branch support was inferred from pseudo-bootstrap with 100 replications, and the tree was rooted at the midpoint (65).

### Antimicrobial activity assays

The pathogenic bacteria used for determining antimicrobial activity include the following: *Staphylococcus aureus* ATCC 25923, methicillin-resistant *Staphylococcus aureus* ATCC 25213, *Bacillus subtilis* ATCC 6633, *Enterococcus faecium* ATCC 51299, *Klebsiella pneumoniae* ATCC 13883, *Escherichia coli* ATCC 25922, *Pseudomonas aeruginosa* ATCC 25924, and *Candida albicans* ATCC 10231. Additionally, the plant pathogen *Ralstonia solanacearum* GIM 1.70 is also included. All strains are preserved in Guangdong Provincial Key Laboratory of Pharmaceutical Bioactive Substances. For extract preparation, strain HD1123-B1^T^ was grown in 300 mL ISP 2 liquid medium (pH 7.2) at 28°C in a rotary shaker (180 rpm) for 14 days. After incubation, the supernatant was separated from the biomass via a high-speed centrifuge at 8,000 rpm for 20 minutes at 4°C. Antimicrobial compounds were recovered from the supernatant by solvent extraction. An equal volume of ethyl acetate was added to the supernatant and mixed vigorously. The organic layer was subjected to evaporation in a rotary vacuum evaporator at 40°C to obtain the crude extract. The crude extract was stored at −20°C for subsequent bioactivity tests.

The disk diffusion method was used to test the antimicrobial activity against the crude extract. LB agar media were heated until melted, cooled to 40°C, and then inoculated with bacterial cells to a final concentration of 0.5 McFarland standard, mixing thoroughly before pouring into Petri dishes by the double-layer plate method. Once the agar solidified, sterile disks (8 mm) were placed on the LB agar inoculated with pathogenic bacteria and on Sabouraud Dextrose Agar (SDA) inoculated with *C. albicans* ATCC 10231. Hundred microliters of the crude extract (10 mg·ml^−1^), positive control, and negative control were added to each disk. The plates were incubated at 37°C for 24 hours for bacteria and at 28°C for 48 hours for fungi. All experiments were repeated in triplicate. The negative control contained only methanol, while the positive controls included vancomycin (256 µg/mL; 512 µg/mL), penicillin (32 µg/mL), ciprofloxacin (8 µg/mL), and amphotericin B (64 µg/mL). Antimicrobial activity was evaluated by measuring the inhibition zones around the disks.

### Mass spectrometric analysis

The fermentation crude extract of strain HD1123-B1^T^ was dissolved in chromatographic methanol to prepare a solution with a concentration of 1 mg/mL. After filtration through a 0.22 µm membrane, the sample was separated using an ultra-high-performance liquid chromatography (UHPLC) system (Thermo Fisher, Waltham, USA) connected to a Q-Exactive-Orbitrap mass spectrometer (Thermo Fisher, Germany). The UHPLC conditions were as follows: A phase was 0.1% formic acid aqueous solution and B phase was methanol. A linear gradient of the mobile phase was applied: from 0 to 5 minutes with 10% B phase for equilibration, then from 5 to 40 minutes, the B phase was linearly increased to 100%, followed by a 10 minute run at 100% B phase. The injection volume was 20 µL, and the flow rate was set at 1 ml/min. The mass spectrometry detection conditions included electrospray ionization in both positive and negative ion modes, with helium as the collision gas and full-scan mode for data acquisition. The mass range for MS^2^ was set between 200 and 1,000. Data collection and processing were performed using Thermo Scientific Xcalibur 4.0 software. The obtained LC-MS/MS data set was converted into mz.XML files by MS Convert software. Finally, the metabolic profile, including the chromatographic peaks, was determined by comparison with the Global Natural Products Social Molecular Networking (GNPS) (https://gnps.ucsd.edu).

## RESULTS AND DISCUSSION

### 16S rRNA phylogenetic analysis

The nearly complete 16S rRNA gene sequence of strain HD1123-B1^T^ (1473 bp) has been deposited in the GenBank/EMBL/DDBJ database under the accession number PP935229. A comparative analysis of this sequence was conducted against type strain sequences using the EzBioCloud server and the NCBI server. Phylogenetic analysis based on the 16S rRNA gene sequence confirmed that strain HD1123-B1^T^ belonged to the genus *Streptomyces*, showing high similarity to *Streptomyces endocoffeicus* CA3R110^T^ (98.80%), *Streptomyces coffeae* CA1R205^T^ (98.47%), and S*treptomyces iranensis* HM35^T^ (97.93%). The similarity values of the 16S rRNA gene sequence of HD1123-B1^T^ obtained from the NCBI server showed consistently that *S. endocoffeicus* CA3R110^T^ (98.88%) represented the closest neighbor to HD1123-B1^T^, followed by *S. coffeae* CA1R205^T^ (98.82%) and *S. iranensis* HM35^T^ (98.76%). Accordingly, these three *Streptomyces* type strains were selected as the closest phylogenetic relatives for further comparative analysis.

**Fig 1:**
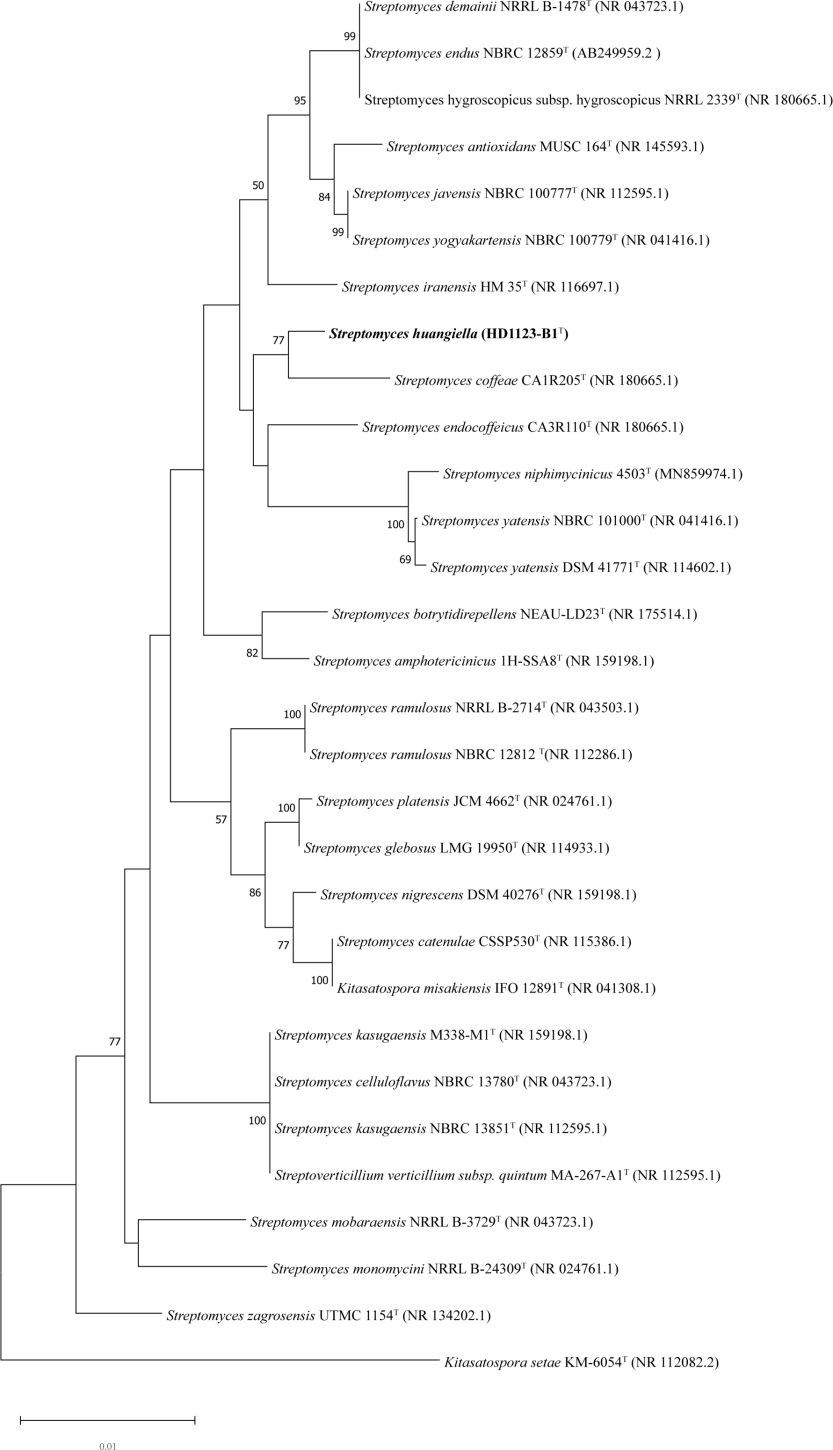
Neighbour-joining phylogenetic tree. Neighbor-joining phylogenetic tree based on 16S rRNA gene sequences, showing the relationships between strains HD1123-B1^T^ and related species of genus *Streptomyces. Kitasatospora setae* KM-6054^T^ (NR_112082.2) was used as an outgroup. Numbers at nodes refer to bootstrap values (based on 1,000 replicates; only values >50% were shown). Bar, 0.01 substitutions per nucleotide position.

Neighbor-joining phylogenetic analysis of the 16S rRNA gene sequence indicated that strain HD1123-B1^T^ forms a distinct cluster with *S. coffeae* CA1R205^T^ (Fig. 1). This clustering was consistently observable in maximum parsimony phylogenetic tree (Fig. S1) and maximum likelihood phylogenetic tree (Fig. S2). Additionally, HD1123-B1^T^ clustered together with *S. endocoffeicus* CA3R110^T^ and *S. iranensis* HM35^T^ in all three phylogenetic analyses, clearly indicating that HD1123-B1^T^ is phylogenetically distinct from these closely related type strains.

### Genome features and genotypic analysis

**Fig 2:**
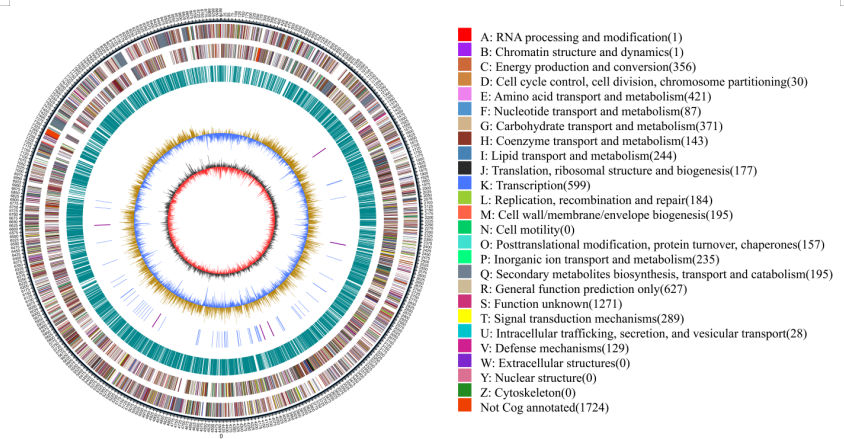
Circular genome map of strain HD1123-B1^T^.The outermost circle marks the size of the genome, with each scale being 5 kb; The second circle and the third circle are the genes on the positive and negative chains of the genome, respectively, and different colors represent different functional classifications of COG. The fourth circle is a repeated sequence. The fifth circle is tRNA and rRNA, blue is tRNA, and purple is rRNA. The sixth circle is the GC content, the light-yellow part indicates that the GC content of this region is higher than the average GC content of the genome; the higher the peak value, the greater the difference between the average GC content, and the blue part indicates that the GC content of this region is lower than the average GC content of the genome. The innermost ring is GC-skew, with dark gray representing regions with more G than C and red representing regions with more C than G.

The assembled genome sequence of strain HD1123-B1^T^ revealed that its genome consisted of a circular chromosome (Fig. 2). The draft genome is 8.9 Mb in size, organized into one scaffold with a contig N50 of 8,879,304 bp, and a coverage of 140.6 X. The DNA G + C content of 71.42 mol% is consistent with that typically observed in the *Streptomyces* genus (66). The genomic analysis identified 7,464 coding sequences, including 73 tRNA genes and 13 rRNA genes. Furthermore, the software tools IslandPath-DIMOB v0.2, CRT v1.2, and PhiSpy v2.3 were employed to predict four genomic islands, four CRISPR regions, and four prophages .The genome sequence of strain HD1123-B1^T^ had been deposited in GenBank under the accession number CP159919. A summary of the general genomic characteristics of strain HD1123-B1^T^ and its closest type strains is provided in Table 1.

**TABLE 1:** Genomic features of *S. huangiella* sp. nov. strains HD1123-B1^T^ and the closest related *Streptomyces* type strains

Feature	*Streptomyces huangiella* sp. nov.	*Streptomyces coffeae* CA1R205^T^	*Streptomyces endocoffeicus* CA3R110^T^	*Streptomyces iranensis* HM 35^T^
Genome size (Mb)	8.9	9.9	13.1	12
Total ungapped length (Mb)	8.9	9.9	13.1	12.0
Number of contigs	1	253	269	82
Contigs N50 (kp)	8879.3	229.8	328.3	327.5
Contigs L50 (kp)	8879.3	12	11	14
DNA G + C percent (mol %)	71.4	70.5	71.0	71.0
Genome coverage (x)	140.6	90.0	138.0	108.0
NCBI accession NO.	PP935229	JAERRF000000000	JAERRG000000000	JAGGLR000000000

The ANI analysis, conducted using OrthoANI software, supports the classification of strain HD1123-B1^T^ as a novel species within the genus *Streptomyces*. The ANI values between HD1123-B1 ^T^ and the reference strains *S. coffeae* CA1R205^T^, *S. endocoffeicus* CA3R110^T^, and *S. iranensis* HM35^T^ were 90.15%, 83.89%, and 83.88%, respectively (Fig. S3), and significantly lower than the species threshold of 95%–96% (67, 68) (Table 2). This finding is consistent with the 16S rRNA phylogenetic analysis, which indicates that strain HD1123-B1^T^ is most closely related to *S. coffeae* CA1R205^T^. Similarly, the dDDH values, calculated using the GGDC algorithm, revealed that the closest species to strain HD1123-B1^T^ is *S. coffeae* CA1R205^T^, with a similarity of 39.3%, well below the 70% cut-off recommended for species delineation (69) (Table 2). The dDDH values between HD1123-B1^T^ and other reference strains, *S. endocoffeicus* CA3R110^T^ and *S. iranensis* HM35 ^T^, were 27.1% and 27.3%, respectively. Based on both ANI and DDH analyses, it is concluded that strain HD1123-B1^T^ represents a new species within the genus *Streptomyces*.

**TABLE 2:** Genome-based comparisons showing the relationship of strains HD1123-B1^T^ with three phylogenetically closely related *Streptomyces* species

Comparison	Strain	*Streptomyces huangiella* sp.nov	*Streptomyces coffeae* CA1R205^T^	*Streptomyces endocoffeicus* CA3R110^T^	*Streptomyces iranensis* HM 35^T^
ANI (orthoANI) (%)	HD1123-B1	100.00	90.15	83.89	83.88
	CA1R205^T^	90.15	100	84.30	84.27
	CA3R110^T^	83.89	84.30	100	94.73
	HM 35^T^	83.88	84.27	94.73	100
dDDH (GGDC) (%)	HD1123-B1	–^*a*^	39.9	27.1	27.3
	CA1R205^T^	39.9	–	27.6	27.7
	CA3R110^T^	27.1	27.6	–	57.0
	HM 35^T^	27.3	27.7	57.0	–

^*a*^ –, No data.

**Fig 3:**
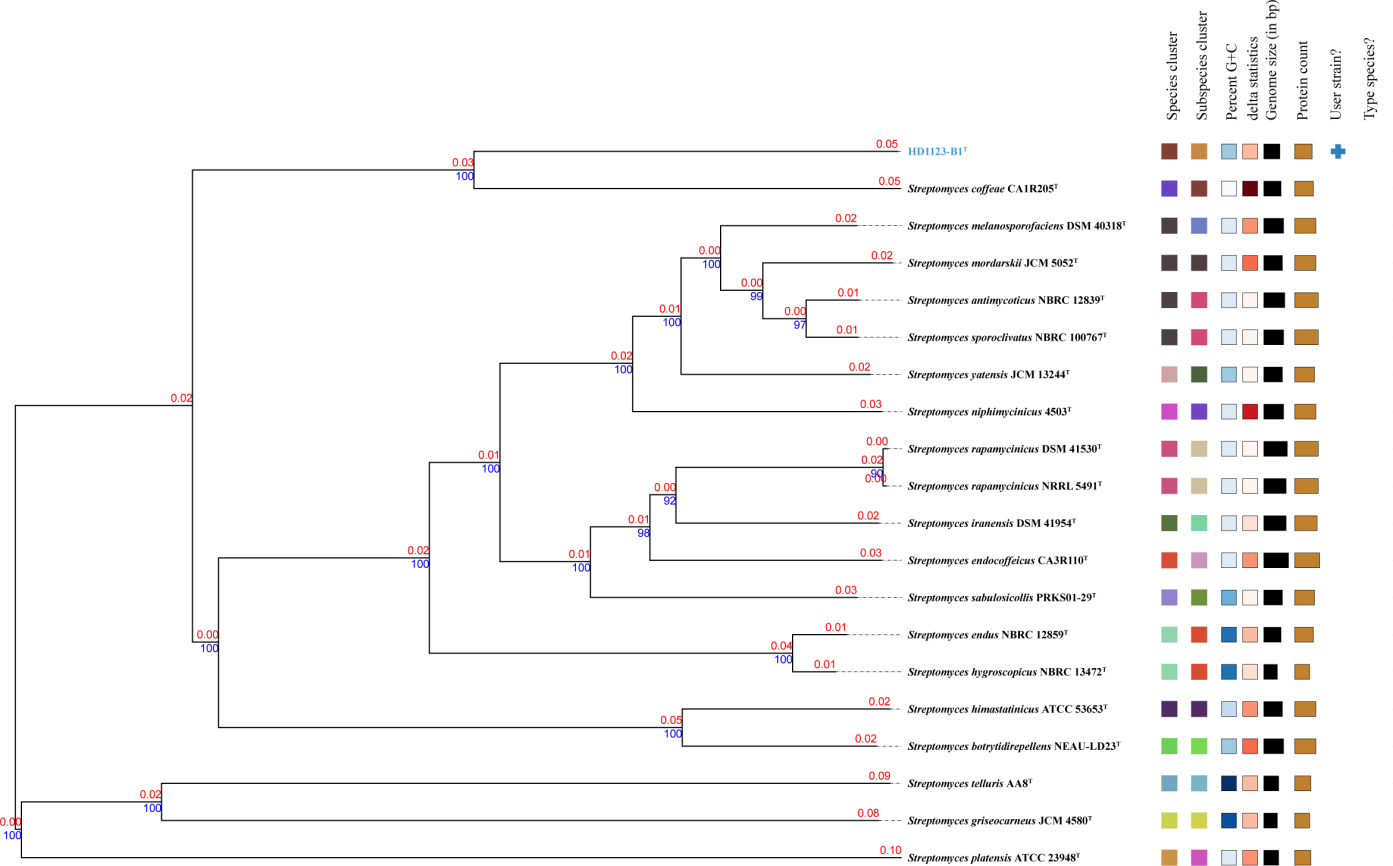
Phylogenomic tree based on genome sequences from strains HD1123-B1^T^ and the closest related type strains in the TYGS database. The tree was inferred with FastME 2.1.6.1 from GBDP distances calculated from genome sequences. The branch lengths are scaled in terms of GBDP distance formula d5. The numbers above branches are GBDP pseudo-bootstrap support values > 60% from 100 replications, with an average branch support of 82.6%. The tree was rooted at the midpoint. The position of the strains of interest is shown in blue.

It is well known that taxa within the genus *Streptomyces* often exhibit high sequence similarity in the 16S rRNA gene, frequently exceeding the ≤97% threshold used for species delineation (70). Therefore, to achieve greater taxonomic resolution, it is advisable to use more robust methods such as whole-genome comparisons and phylogenomic analyses when assessing potential new *Streptomyces* species. To further clarify the phylogenetic position of strain HD1123-B1^T^, a phylogenomic tree was constructed using FASTME 2.1.6.1, which revealed that strain HD1123-B1^T^ forms an independent clade with *S. coffeae* CA1R205^T^, supported by high bootstrap values of 100% (Fig. 3). This phylogenomic analysis further confirmed that HD1123-B1^T^ is most closely related to the type strains within the genus *Streptomyces*, while other reference strains, such as *S. endocoffeicus* CA3R110^T^ and *S. iranensis* HM35^T^, are more distantly related. Collectively, these data strongly indicate that strain HD1123-B1^T^ belongs to a new species within the genus *Streptomyces*.

### Morphological, physiological, and biochemical characteristics

Although genomic analysis has confirmed the taxonomic status of HD1123-B1^T^ as a novel species of the genus *Streptomyces*, a detailed analysis of phenotypic characteristics is required to substantiate its novelty. Based on the 16S rRNA gene similarity and the phylogenetic placement, closely related type strains *S. coffeae* CA1R205^T^, *S. iranensis* HM35^T^, and *S. endocoffeicus* CA3R110^T^ were selected for phenotypic comparison.

**Fig 4:**
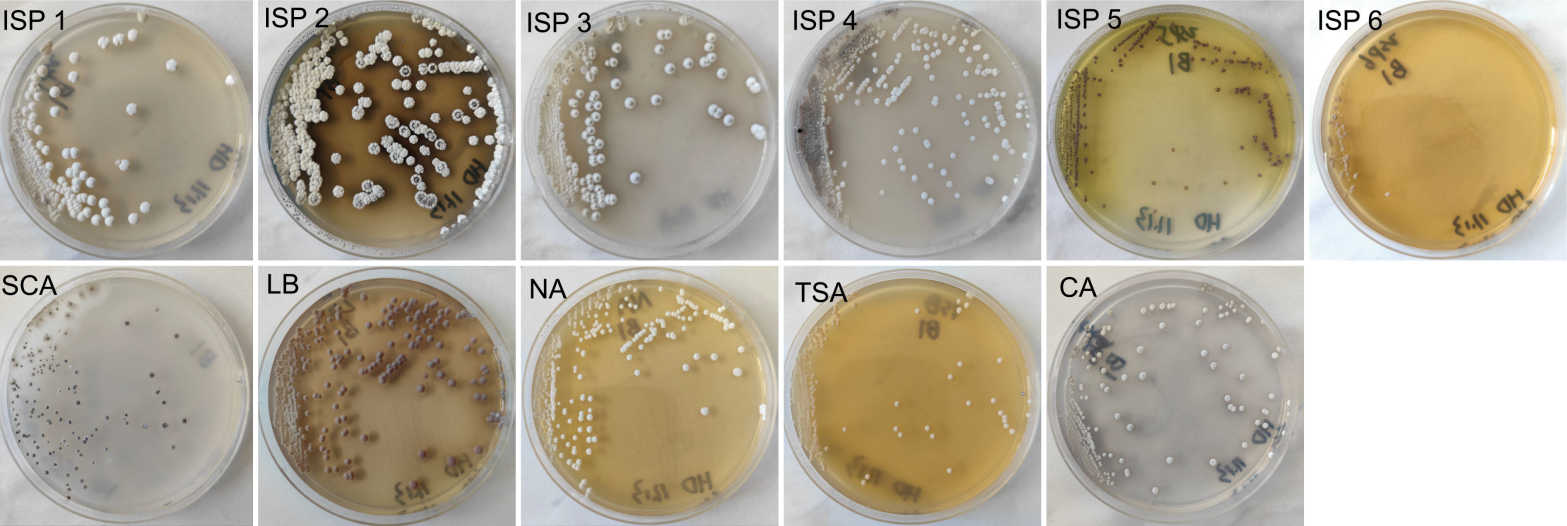
Culture characteristics of the strain on various media.

Strain HD1123-B1^T^ is a gram-positive bacterium (Fig. S4), characterized by circular colonies with a convex surface, medium size, and smooth edges. The colonies exhibited "agar eating" phenomena, producing a white aerial mycelium without pigment. These cultural and morphological traits are consistent with those typical of the genus *Streptomyces*. To clarify the novelty of strain HD1123-B1^T^ at the species taxonomic level, a comparative analysis of phenotypic characteristics between strain HD1123-B1^T^ and its reference strains was conducted (Table S1). The substrate mycelium and aerial mycelium displayed distinct colors on various media, with notable differences in colony coloration compared to its closest phylogenetic relatives. The substrate mycelium of HD1123-B1^T^ appeared brownish black on ISP 2 medium, while the substrate mycelia of *S. coffeae* CA1R205^T^, *S. endocoffeicus* CA3R110^T^, and *S. iranensis* HM35^T^ were dark brownish black, pale greenish yellow, and beige, respectively. HD1123-B1^T^ exhibited robust growth on ISP 2, ISP 3, ISP 4, LB, NA, and CA media, forming a well-developed substrate mycelium. In contrast, slower growth was observed on ISP 1, ISP 5, and TSA media, with poor growth on ISP 6, and no melanin production was detected on ISP 6. The substrate mycelium ranged in color from yellowish-white to brownish-black, while the aerial mycelium was white on ISP 1, ISP 2, ISP 3, ISP 4, NA, and CA media, with abundant aerial mycelium on ISP 2 agar. Dark grayish-brown soluble pigments were observed on ISP 2 agar, and strong yellow-green soluble pigments on ISP 5 agar; no pigment production was noted on other media (Fig. 4). *S. coffeae* CA1R205^T^ and *S. endocoffeicus* CA3R110^T^ did not produce any pigmentation on ISP 2 medium, while *S. iranensis* HM35^T^ produced pale yellow pigmentation on ISP 2 medium. On ISP 5 medium, *S. coffeae* CA1R205^T^ and *S. endocoffeicus* CA3R110^T^ produced light yellowish-brown and brilliant greenish-yellow pigments, respectively, whereas *S. iranensis* HM35^T^ did not produce any pigmentation on ISP 5 medium.

SEM revealed that HD1123-B1^T^ produced abundant substrate and aerial mycelium, characterized by long, dense, multibranched straight strips with smooth surfaces and warts (Fig. S5), without spiral chains of spores. This morphology is consistent with members of the genus *Streptomyces* (8, 71). In contrast, *S. coffeae* CA1R205^T^, the most closely related strain, produced spiral chains of spores with a wrinkled surface and was nonmotile (72). Similarly, *S. endocoffeicus* CA3R110^T^ also produced spiral chains of spores with a wrinkled surface and was nonmotile (73). *S. iranensis* HM35^T^ possessed short and dense spiral chains of spores and wrinkled spores (74). These differences are key to distinguishing strain HD1123-B1^T^ from the reference strains.

In addition to morphological and cultural characteristics, physiological and biochemical features of strain HD1123-B1^T^ were also examined to distinguish it from other *Streptomyces* taxa (Table 3). Strain HD1123-B1^T^ grown at a pH range of 6–9 (optimal at 7), NaCl concentrations of 0%–2% (wt/vol), and at a temperature range of 20%–40°C, with an optimal growth temperature of 28°C, respectively. These tolerance ranges were similar to those of reference strains. However, strain HD1123-B1^T^ could grow at 2% (wt/vol) NaCl but failed to grow at 3% (wt/vol) NaCl, whereas *S. coffeae* CA1R205^T^, *S. endocoffeicus* CA3R110^T^, and *S. iranensis* HM35^T^ could grow at 3% (wt/vol) NaCl. HD1123-B1^T^ utilized D-galactose, lactose, maltotriose, myo-inositol, D-ribose, inulin, trehalose, mannose, and salicylin as sole carbon sources but could not utilize L-arabinose, D-mannitol, L-rhamnose, cellobiose, sucrose, raffinose, D-glucose, D-fructose, dextran, or glycerol. Additionally, the strain showed positive activity for catalase, citrate utilization, lipase hydrolysis (Tween 20, 60, and 80), casein hydrolysis, and esculin, while testing negative for oxidase, milk coagulation and peptonization, starch hydrolysis, nitrate reduction, urease activity, hippurate hydrolysis, tryptophan degradation, hydrogen sulfide production, and gelatin liquefaction. Most characteristics observed in this study were consistent with those described for the three reference strains, including maximum growth temperature (40℃), growth pH range (6–9), and negative results for gelatin liquefaction and urease activity. Both HD1123-B1^T^ and its three reference strains could utilize maltotriose, D-ribose, mannose, inulin, trehalose, and myo-inositol but could not utilize sucrose, arabinose, L-rhamnose, cellobiose, or glycerol. The strains HD1123-B1^T^ were positive for the utilization of galactose, salicylin, and citrate, and hydrolyzed escin and Tween (20, 59, 75), which were negative in the descriptions of the three reference strains. The strain tested positive for dextran, xylitol, xylose, D-ribose, and mannose in the carbon source utilization test, as well as for the oxidase test and milk coagulation test. In contrast, the results were opposite to those of the closely related strain *S. coffeae* CA1R205^T^, indicating significant differences in their physiological and biochemical characteristics.

**TABLE 3:** Differential characteristics of HD1123-B1^T^ and its closest phylogenetic relatives. All phenotypic data were determined in this study^*a*^

Characteristics	*Streptomyces huangiella* sp. nov	Streptomyces coffeae CA1R205^T^	*Streptomyces endocoffeicus* CA3R110^T^	Streptomyces iranensis Hm 35^T^
Growth with/at:				
Maximum temperature for growth (℃)	40	40	40	37
pH range for growth	6–9	6–9	6–9	6–10
Maximum NaCl tolerance (%wt/vol)	2	3	3	3
Color of substrate mycelium on ISP 2(14 days):				
Color of aerial mycelium	White	N	Grayish-white	Grayish-white to Gray
Color of substrate mycelium	Brownish black	Dark brownish black	Pale greenish yellow	Brownish yellow
Coagulation of milk	−	+	+	+
Gelatin liquefaction	−	−	+	+
Urease activity	−	−	+	−
Carbon source utilization 1% (wt/vol):				
Lactose	w	+	w	+
Melibiose	+	+	+	+
Inositol	+	+	+	+
Dextran	−	+	+	−
Xylitol	−	+	+	−
D-Xylose	−	+	−	+
L-Arabinose	−	−	−	+
D-Mannitol	-	-	+	+
D-Ribose	+	-	-	+
D-Mannose	+	-	+	+
L-Rhamnose	-	-	+	+
Raffinose	-	-	-	+
Sucrose	-	-	-	+
Glycerol	-	-	-	+
Hydrolysis of:				
L-tyrosine	+	-	+	+
Cellulose	−	-	-	N

^*a*^ +, Positive; −, Negative; w, Weakly positive; N, No data.

Based on genotypic and phenotypic characteristics, strain HD1123-B1^T^ is confirmed as a novel species of the genus *Streptomyces*, for which the name *Streptomyces huangiella* sp. nov. is proposed.

### Genome annotations and analysis

**Fig 5:**
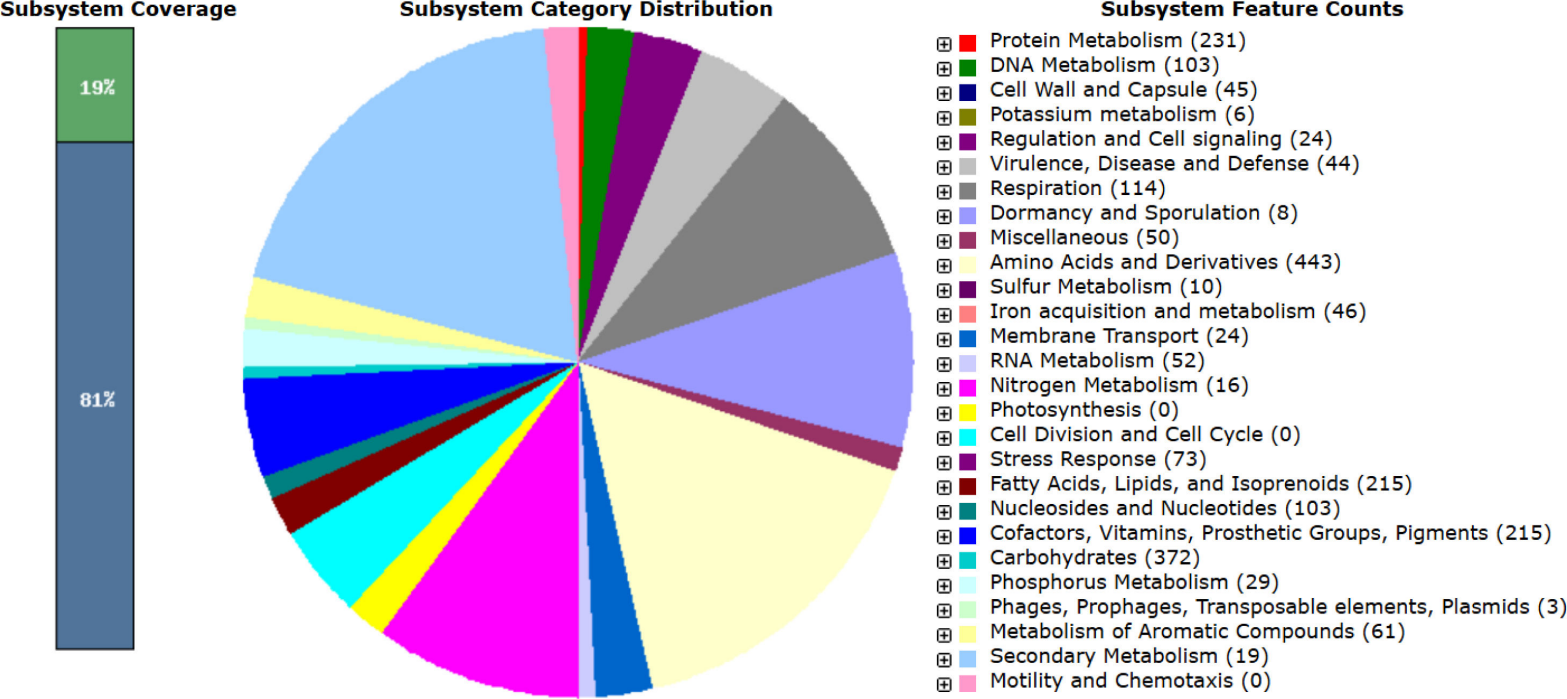
Subsystem category distribution of strain HD1123-B1^T^ based on the RAST annotation server.

According to the RAST predictions using the SEED viewer, strain HD1123-B1^T^ was distributed across 333 subsystems. The majority of the annotated genes were associated with Amino Acids and Derivatives (19.2%), Carbohydrates (16.1%), Protein Metabolism (10.0%), Cofactors, Vitamins, Prosthetic Groups, and Pigments (9.3%), and Fatty Acids, Lipids, and Isoprenoids (9.3%). Notably, genes related to secondary metabolism accounted for only 0.8% of the genome (Fig. 5).

Additionally, 5,740, 5,034, and 2,567 genes were annotated using the eggNOG, GO, and KEGG databases, respectively. In the eggNOG classification, the genes were primarily involved in transcription (10.28%), amino acid transport and metabolism (7.22%), carbohydrate transport and metabolism (6.61%), energy production and conversion (6.11%), and signal transduction mechanisms (5.03%). Interestingly, 21.81% of the genes had unknown functions (Fig. S6a). The KEGG pathway analysis revealed that the genome of strain HD1123-B1^T^ included genes encoding ABC transporters, as well as those involved in carbon metabolism; biosynthesis of amino acids; fatty acid metabolism,;glycine, serine, and threonine metabolism; and the citrate cycle (TCA cycle) (Fig. S6b). Furthermore, strain HD1123-B1^T^ possessed pathways for phenylalanine metabolism, methane metabolism, degradation of aromatic compounds, and benzoate degradation, suggesting its potential to utilize these organic compounds for energy metabolism. The GO functional annotation revealed that the strain had 2,933 genes associated with catalytic activity, 2,399 with metabolic processes, 769 with biological regulation, and 142 genes coding for macromolecular complexes with specific biological functions (Fig. S6c).

### Antibacterial activity of strains HD1123-B1^T^

**Fig 6:**
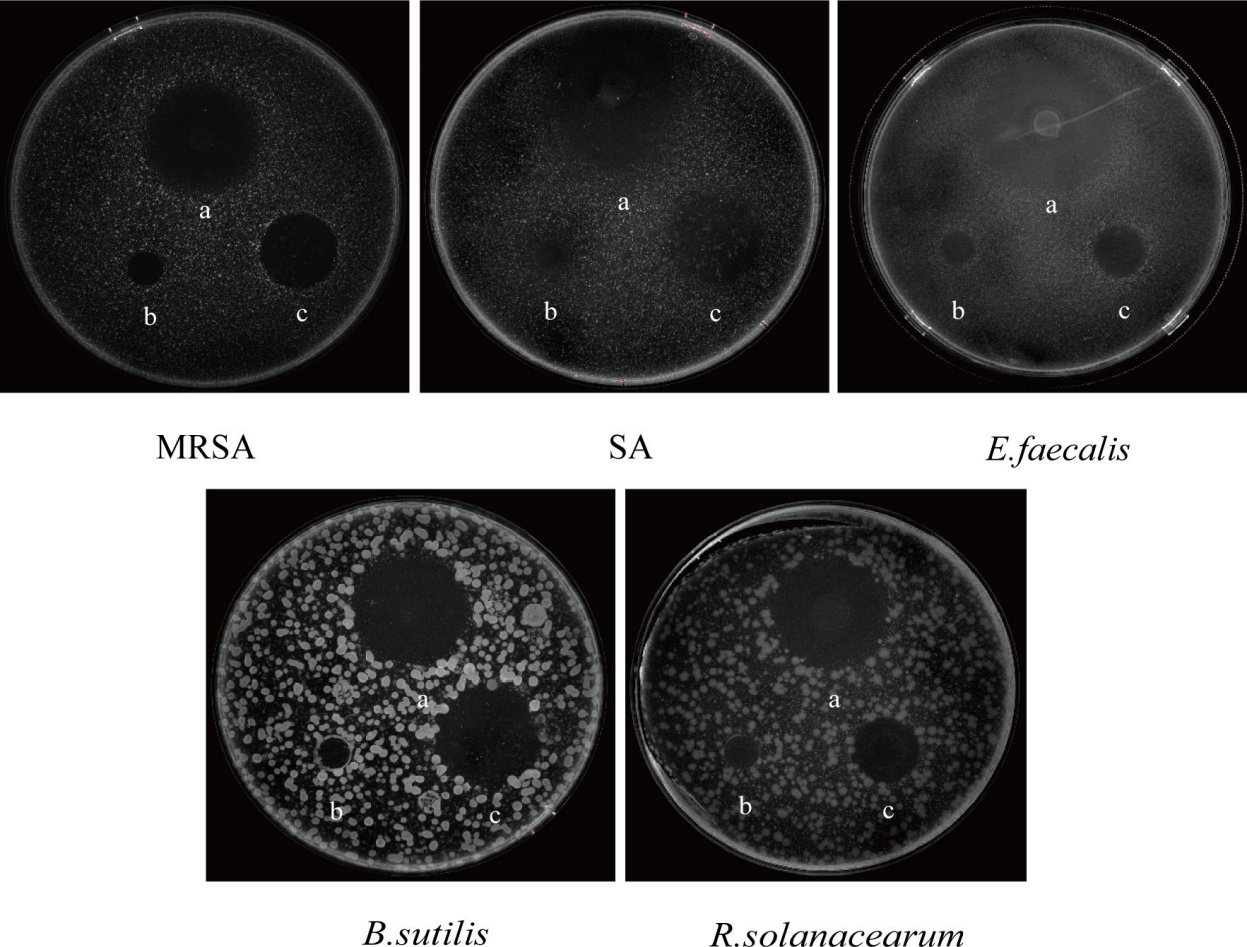
The inhibitory zone size of the crude extract of the strain HD1123-B1^T^ against pathogenic bacteria. (a, the crude extract; b, the negative control; c, the positive control).

The antibacterial assays revealed that the crude extract of strain HD1123-B1^T^ exhibited significant inhibitory effects against several gram-positive bacteria after 12 hours of cultivation, including *S. aureus* ATCC 25923, MRSA ATCC 25213, *B. subtilis* ATCC 6633, and *E. faecalis* ATCC 51299, and its inhibitory zones were 23.80, 24.18, 26.68, and 26.30 mm, respectively. In contrast, the strain showed no antibacterial activity against the gram-negative bacteria *K. pneumoniae* ATCC 13883, *E. coli* ATCC 25922, *P. aeruginosa* ATCC 25924, or against the fungus *C. albicans* ATCC 10231. Additionally, the strain was tested against the plant pathogenic bacterium *R. solanacearum* GIM 1.70 to assess its potential as an agricultural biocontrol agent. Notably, HD1123-B1^T^ demonstrated significant inhibitory activity against *R. solanacearum* GIM 1.70, and its inhibitory zone was 24.80 mm, respectively (Table S2). These results indicated that strain HD1123-B1^T^ had tremendous potential for producing secondary metabolites that exhibit inhibitory activity against human and plant pathogens. The complete antibacterial activity profiles of each strain are shown in Fig. 6.

### Biosynthetic gene cluster and metabolome combine analysis

Using antiSMASH V8.0 for genomic mining of potential secondary metabolite-related biosynthetic gene clusters (BGCs), the results indicated that strain HD1123-B1^T^ possesses 38 BGCs encoding secondary metabolites (refer to Table 4). These BGCs were responsible for the biosynthesis of known and/or novel secondary metabolites, including nine type I polyketide synthases (T1PKS), five non-ribosomal peptide synthetase (NRPS), five terpene, three siderophore, two RiPP-like, two ladderane, one butyrolactone, one type II polyketide synthases (T2PKS), one lanthipeptide-class-iii, one NRPS-like, one indole, one thioamitides, one RRE-containing, one thiopeptide, one ectoine, one lanthipeptide-class-i, one melanin, and one LAP. Common secondary metabolite BGCs identified in the genus *Streptomyces*, such as streptobactin, hopene, 2-methylisoborneol, melanin, and ectoine, were found to be present in the strain HD1123-B1^T^. The predicted BGCs from strain HD1123-B1^T^ were primarily classified as NRPSs, T1PKSs, and terpene, which represent dominant biosynthetic pathways in many actinobacteria genomes known to produce various bioactive compounds (33). Five predicted BGCs showed high similarity (>70%) to the streptobactin, elaiophylin, spore pigment, echoside A, and isorenieratene gene clusters. Six predict BGCs exhibited moderate similarity (30-70%) to hopene, catenulipeptin, atratumycin, 5-isoprenylindole-3-carboxylate β-D-glycosyl ester, atratumycin, and bafilomycin B1 BGC. Sixteen predicted BGC had lower similarity (<30%) with A54145, rimosamide, salinomycin, surugamide A, colabomycin, granaticin, A-503083 A, lugdunomycin, herbimycin A, kosinostatin, niphimycins C–E, akaeolide, melanin, funisamine, streptovaricin, and herboxidiene gene clusters. Notably, five predicted BGCs showed 100% identity with desferrioxamine B, geosmin, ectoine, 2-methylisoborneol, and nigericin. Deferoxamine (deferoxamine B) is a natural iron chelator (combined with Fe(III) and many other metal cations) widely used to reduce iron accumulation and deposition in tissues. Deferoxamine exhibits notable antioxidant activity and can upregulate HIF-1α levels (76). It also possesses anticancer properties by extracting iron ions from cancer cells, inducing apoptosis and autophagy (77). Ectoine is a compatible solute derived from halophilic bacteria (*Halomonas elongata*). Under extreme conditions such as high salinity, high temperature, and high ultraviolet radiation, ectoine protects halophiles from damage. Pristinol, a sesquiterpene alcohol, possesses an unusual skeleton derived from the actinobacterium *Streptomyces pristinaespiralis* (78). The terpenoid compounds geosmin and 2-methylisoborneol are produced by actinomycetes, cyanobacteria, myxobacteria, and fungi (79). These compounds do not exhibit any antibacterial activity. Nigericin is a polyether antibiotic, a very important natural product produced by *Streptomyces hygroscopicus* (25). Current studies have reported that it possesses antibacterial, antifungal, antimalarial, and anticancer activities, showing significant bactericidal effects against various drug-resistant gram-positive bacteria (26, 27, 75). The above results highlight the genomic potential of these new strains in the discovery of natural products.

**TABLE 4:** BGCs in *Streptomyces huangiella* HD1123-B1^T^ predicted by AntiSMASH v8.0^*a*^

Cluster	Type	Location in the S. *huangiella* HD1123-B1^T^ genome (nt)	Specialized metabolite encoded by a predicted BGC	Similarity
1	T1PKS	184,685–227,112	A54145	5%
2	NRPS, linaridin	414,204–462,565	Rimosamide	21%
3	NRPS	563,919–610,815	Streptobactin	76%
4	T1PKS, RiPP-like	767,488–811,979	Salinomycin	6%
5	T1PKS	905,958–984,794	Elaiophylin	83%
6	Butyrolactone	905,958–984,794	NA	
7	NRPS	1,207,164–1,285,428	Surugamide A, D	19%
8	Terpene	1,436,563–1,456,061	Pristinol	100%
9	RiPP-like	1,806,920–1,816,820	NA	
10	Terpene	1,869,145–1,895,060	Hopene	69%
11	T2PKS	1,978,159–2,050,665	Spore pigment	75%
12	RiPP-like	2,250,769–2,260,694	NA	
13	Siderophore	2,432,112–2,442,680	NA	
14	Lanthipeptide-class-iii	2,759,702–2,782,317	Catenulipeptin	60%
15	NRPS-like	2,974,252–3,014,013	Echoside A ~ E	88%
16	Siderophore	3,454,557–3,465,883	Desferrioxamine B	100%
17	Terpene	4,431,683–4,453,088	Geosmin	100%
18	Ladderane	4,525,442–4,566,096	Colabomycin	20%
19	NRPS, NRPS-like, arylpolyene, ladderane	4,726,776–4,824,192	Atratumycin	55%
20	Indole	5,142,470–5,163,627	5-isoprenylindole-3-carboxylate β-D-glycosyl ester	61%
21	Thioamitides	5,354,927–5,377,586	NA	
22	Arylpolyene, ladderane	5,433,084–5,475,458	Atratumycin	34%
23	RRE-containing	5,480,765–5,501,849	Granaticin	10%
24	Other, thiopeptide	6,044,718-6,103,294	A-503083 A, B, E, F	7%
25	Ectoine	6,513,226–6,523,225	Ectoine	100%
26	Siderophore	6,686,695–6,700,431	Lugdunomycin	11%
27	Terpene	6,978,303–6,997,057	2-methylisoborneol	100%
28	Lanthipeptide-class-i	7,057,744–7,082,887	Herbimycin A	20%
29	NRPS, T1PKS, ectoine	7,107,029-7,219,137	kosinostatin	11%
30	T1PKS, hglE-KS	7,346,646-7,462,955	niphimycins C-E	29%
31	T1PKS, butyrolactone	7,477,178-7,525,748	akaeolide	16%
32	melanin	7,610,312-7,620,752	melanin	28%
33	RiPP-like, terpene	7,651,748-7,681,930	isorenieratene	85%
34	T1PKS	7,743,024-7,791,575	funisamine	20%
35	T1PKS	8,041,613-8,172,620	nigericin	100%
36	T1PKS	8,378,959–8,475,070	Bafilomycin B1	66%
37	T1PKS	8,552,369–8,640,780	Streptovaricin	12%
38	LAP	8,655,699–8,694,193	Herboxidiene	4%

^*a*^ NA, Not available.

Based on BGC analysis and comparisons to known databases, the BGC Cluster 35 of HD1123-B1^T^ showed a structural similarity of 100% to the nigericin biosynthetic gene cluster identified in *Streptomyces violaceusniger* DSM4137^T^ (80). This cluster contained a core biosynthetic gene, GE00688, which encodes malonyl CoA-acyl carrier protein transacylase (homologous to ABC84461.1 from *S. violaceusniger*), along with eight other core biosynthetic genes (GE006882, GE006883, GE006884, GE006885, GE006886, GE006894, GE006895, and GE006896) that encode beta-ketoacyl synthases. Malonyl CoA-acyl carrier protein transacylase (MCAT) is a critical enzyme responsible for the initiation step of type II fatty acid synthesis (FAS II) in bacteria, which transfers the malonyl moiety to the holo-acyl carrier protein (ACP), forming malonyl-ACP intermediates (81). MCAT had also been reported to be involved in polyketide biosynthesis, a class of structurally diverse bioactive secondary metabolites, such as tetracyclines and erythromycin (82). Therefore, MCAT is considered an important enzyme in bacterial metabolic activity and an attractive drug target for the discovery of antimicrobial agents. Beta-ketoacyl synthase is a key enzyme involved in the biosynthesis of various natural products, including fatty acids, polyketide precursors for commercially important pharmacological agents, and mycolic acid precursors in pathogenic mycobacteria. These enzymes exhibit a wide range of sequence similarities and catalyze the formation of new carbon-carbon bonds through the condensation of various acyl chain precursors with extending carbon sources (typically malonyl or methylmalonyl moieties), which are covalently linked to ACP via thioester bonds (83). Beta-ketoacyl synthase has been identified as a target for the development of new anticancer drugs (84). Additional biosynthetic genes GE006880, GE006887, GE006888, GE006889, and GE006891 encode proteins homologous to methyltransferase, haloalkane dehalogenase, cytochrome P450, phosphopantetheine-binding domain-containing protein, and monooxygenase FAD-binding, respectively. Nigericin is a polyether produced by various *Streptomyces* strains that can bind monovalent or polyvalent cations, forming lipophilic complexes capable of transporting metal cations across cell membranes, leading to loss of membrane integrity and subsequent cell death (85). Current studies have confirmed that nigericin disrupts the cell membrane of gram-positive bacteria, increasing permeability; additionally, it exerts antimicrobial activity by reducing positive charges on the cell membrane through the depletion of the GraSR two-component regulatory system, resulting in strong bactericidal effects against clinically multidrug-resistant gram-positive bacteria (27).

**Fig 7:**
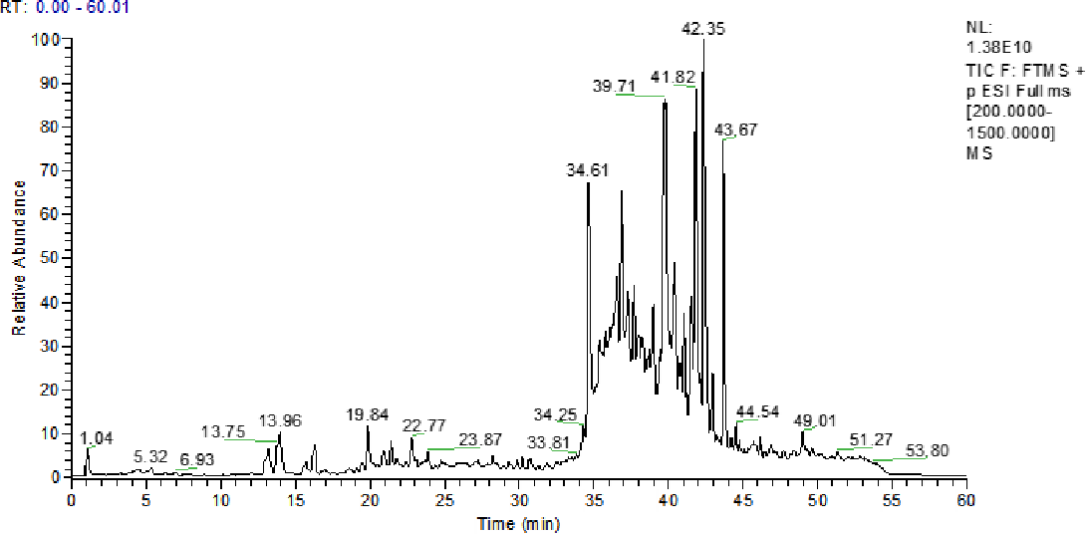
Chromatogram of LC–MS/MS of the ethyl acetate extract of the strain HD1123-B1^T^.

**Fig 8:**
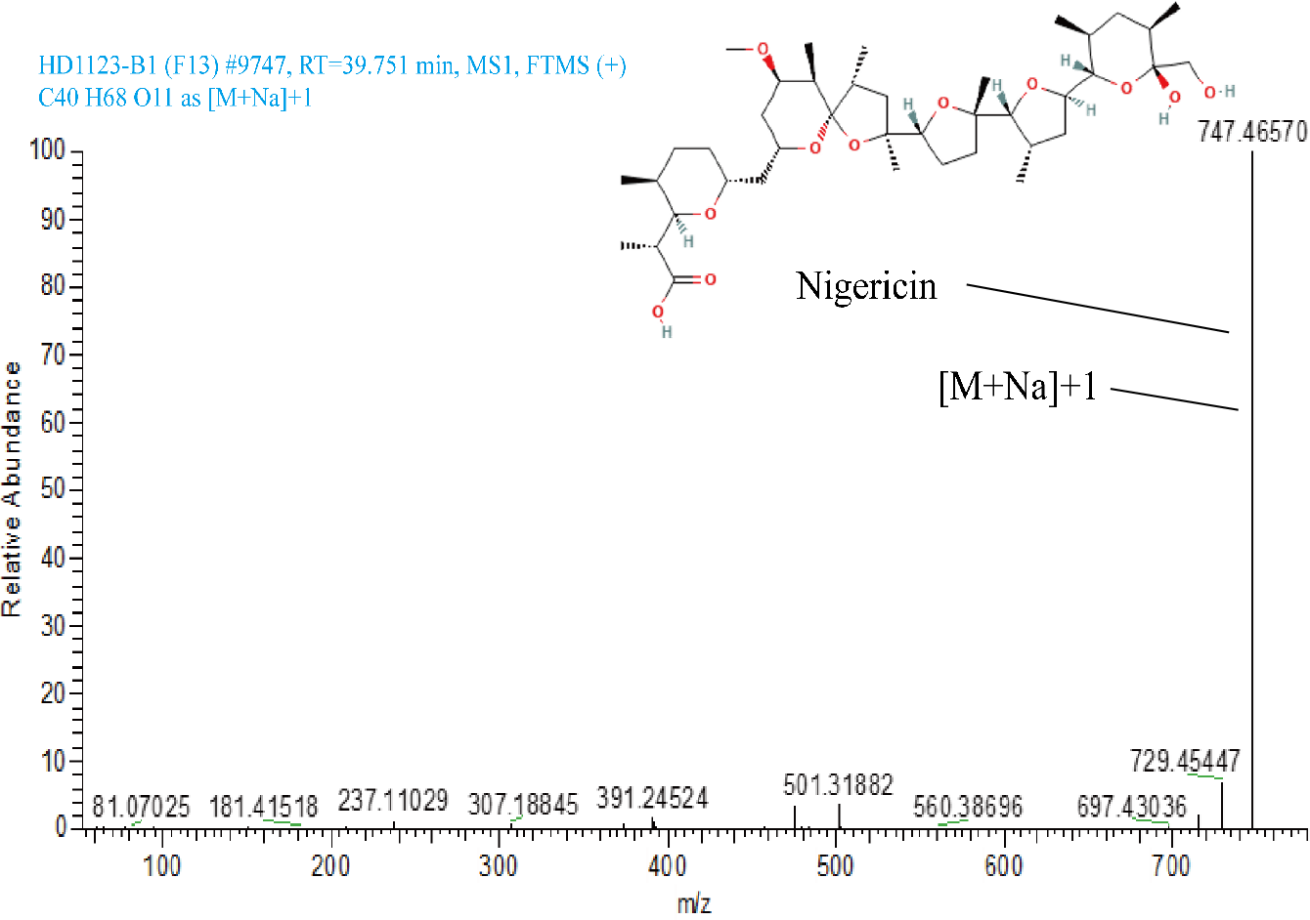
High-resolution LC-MS/MS analysis of the ethyl acetate (EA) extract of Nigericin in the fermentation broth of *Streptomyces huangiella* HD1123-B1^T^. The Nigericin (RT time :39.71, [M + H] ^+^ at *m/z* 724.5).

The ethyl acetate extract from the fermentation broth of strain HD1123-B1^T^ was studied by LC-MS/MS to test the hypothesis that the strain could produce predicted bioactive secondary metabolites. The mass spectrometry data were obtained in the positive mode. The total ion currents of LC-MS/MS in the positive ion modes of the crude extract of strain HD1133-B1^T^ are shown in Fig. 7. Metabolites in the fermentation broth were identified based on the mass-to-charge ratios (m/z) of molecular ions from the UPLC-MS/MS spectrum. A comparison of the secondary metabolite profile of the strain with genomic mining data https://www.ezbiocloud.net/tools/ani confirmed the biosynthesis of nigericin, which had a molecular formula of C_40_H_68_O_11_, a retention time of 39.71 minutes, and an *m/z* value of 724.5 (Fig. 8), respectively, aligned with nigericin isolated from the secondary metabolites of *Streptomyces hygroscopicus*.

This study found that the ethyl acetate crude extract of strain HD1123-B1^T^ exhibited significant antibacterial effects against four gram-positive bacteria: *S. aureus* ATCC 25923, *Methicillin-resistant S. aureus* ATCC 25213, *B. subtilis* ATCC 6633, and *E. faecalis* ATCC 51299. These results aligned with the findings of Huang et al. (86), which could be inferred that the strain inhibited the growth of gram-positive bacteria and resistant strains through the production of nigericin to achieve a bactericidal effect. Furthermore, the ethyl acetate crude extract also demonstrated unexpectedly strong antibacterial activity against *R. solanacearum* GIM 1.70 (a gram-negative bacterium). Nigericin shows weak antibacterial effects against gram-negative bacteria, significantly lower than its activity against gram-positive bacteria, primarily due to the unique outer membrane barrier and efflux mechanisms of gram-negative bacteria. Currently, there are few studies reporting significant antibacterial activity and mechanisms of nigericin against gram-negative bacteria. The ethyl acetate crude extract of strain HD1123-B1^T^ demonstrated inhibitory effects against gram-negative bacteria (87), possibly because nigericin antagonizes other secondary metabolites produced by the strain itself. Research by Taechowisan T et al. (88) found that the compounds 1-methyl ester-nigericin (1) and methyl 5-(hydroxymethyl) furan-2-carboxylate (2) have synergistic potential as antibacterial agents against infections caused by *Proteus* spp. with low cytotoxicity. The MIC ranges for compounds 1 and 2 were 39.06–78.12 μg/mL and 78.12–156.25 μg/mL, respectively, and when combined, the MIC values were lower than those of the individual tested compounds (FICI = 0.28–0.50). Moreover, these compounds exhibited low cytotoxicity when used in combination and inhibited biofilm formation of *Proteus* spp. Meanwhile, research by Amit Kumar Sahu et al. (89) revealed that the black mycelium produced by a novel *Streptomyces* strain DASNCL-29 displayed polymorphism in its crystal structure, forming monoclinic and orthorhombic lattices when crystallized with methanol and hexane, respectively. Fluorinated analogs NIG-3 and NIG-5 acted on cells, causing lysis and thus inhibiting the growth of the gram-positive bacterium *Staphylococcus aureus* ATCC 9144 and the gram-negative bacterium *Escherichia coli* ATCC 8739. Therefore, nigericin may undergo chemical modification during the preparation of crude extracts to form fluorinated analogs, or the strain might produce other bioactive secondary metabolites, which could exert strong antibacterial activity against gram-negative bacteria.

Based on genomic and metabolomic analyses, the strain HD1123-B1^T^ has been confirmed to produce the bioactive compound nigericin, which may represent a promising source for discovering bioactive metabolites against multidrug-resistant pathogens.

### Conclusion (includes descriptions)

A new strain with significant antibacterial activity, with HD1123-B1^T^ (=GDMCC 4.423^T^ =JCM 37538^T^) designated as the type strain, was isolated from the gut contents of *Pheretima aspergillum* collected in Guangzhou, China. A comprehensive analysis using a polyphasic taxonomic approach demonstrated significant novelty in the morphological, physiological, biochemical, and genomic characteristics of this strain. Phylogenetic analysis revealed that the 16S rRNA gene sequence of HD1123-B1^T^ shares only 98.47% similarity with the closely related species *S. coffeae* CA1R205^T^, which is below the species delineation threshold of 98.70%. Comparative genomic analysis confirmed that the ANI and dDDH values between this strain and *S. coffeae* CA1R205^T^ are 90.15% and 39.9%, respectively, both significantly lower than the recognized species classification standards. Whole-genome sequencing revealed that HD1123-B1^T^ contains 38 BGCs, with 42% of the BGCs showing less than 30% similarity, suggesting a rich potential for the synthesis of novel bioactive compounds. Notably, antibacterial assays demonstrated that the fermentation products of this strain exhibited significant inhibitory activity against various gram-positive bacteria, outperforming commonly used clinical antibiotics. Furthermore, it showed promising biocontrol potential against the plant pathogen *Ralstonia solanacearum* GIM 1.70. Through genomic mining and metabolomics analysis, we identified a complete biosynthetic gene cluster for nigericin in this strain and confirmed its ability to produce nigericin. This research not only enriches the species diversity within the *Streptomyces* genus but also provides important resources for the development of novel bioactive compounds.

Based on these findings, future work will focus on the detailed genomic-guided isolation and structural identification of the products from these biosynthetic gene clusters, as well as the construction of nigericin biosynthetic mutants of *Streptomyces huangdansis* sp. nov. to further evaluate its potential clinical application value. Table 5 provides the formal description of this new species, and its type strain has been deposited in the GDMCC and JCM culture collections.

**TABLE 5:** Descriptions of *Streptomyces huangiella* sp. nov

Genus’s name	Streptomyces
Species name	*Streptomyces huangiella*
Specific epithet	*huangiella*
Species status	sp. nov
Species etymology	Hu.ang’i.ella. N.L. fem. gen. n. huang of the people, who isolated the type strain firstly
Description of the new taxon and diagnostic traits	Cells are gram-stain-positive, aerobic, mycelium-forming, and filamentous. The substrate mycelium is brownish black on ISP two agar. Colonies on Gause’s Synthetic Agar are circular, convex, neat edge, concentric, and producing white aerial mycelium without pigment production. Cells grew well on ISP 1-5 agar and formed abundant substrate mycelium. White aerial mycelium is formed on ISP 1, ISP 2, ISP 3, ISP 4, Nutrient Agar, and Czapek’s Dox Agar. Dark grayish-brown to light yellowish-brown diffusible pigments were detected on ISP 2 and ISP 5 agar. Does not produce melanin on ISP 6. SEM showed that substrate mycelium and aerial mycelium were abundant, substrate mycelium was long, dense, and multi-branched, spore filaments were straight, smooth, and wart-like, and no spiral chains of spores with rugose surfaces were observed. Cell growth was observed in the range of 20–40℃ and at pH 6–9. Maximum NaCl for growth is 3% (wt/vol). Optimal growth conditions occur at pH 7 and 28 ℃. Capable of utilizing d-galactose, lactose, meliose, inositol, D-ribose, inulin, trehalose, mannose, and salicin as sole carbon sources but cannot utilize L-arabinose, D-mannitol, L-rhamnoose, raffinose, sucrose, cellobiose, D-glucose, d-fructose, dextran, or glycerol. Positive for catalase. Degrades tweens (20, 60, and 80), citrate, casein, and esculoside, but not tween 20, starch, tryptophan, and hippurate. Negative for oxidase, milk coagulation, nitrate reduction, H2S production, gelatin liquefaction, peptonization, and urease activity.
Country of origin	China
Region of origin	Guangzhou City
Date of isolation	10/2023
Source of isolation	The gut contents of the *Pheretima aspergillum*
Sampling date	06/2023
Latitude	N 23.055278°
Longitude	E 113.408818°
16S rRNA gene accession number	PP935229
Genome accession number	CP159919
Genome status	Draft genome sequence
Genome size	8.9 Mb
DNA G + C content (%)	71.42
Number of strains In study	1
Designation of the Type Strain	HD1123-B1^T^
Strain Collection Numbers	=GDMCC 4.423^T^ =JCM 37538^T^

## Data Availability

The type strain, HD1123-B1^T^ (=GDMCC 4.423^T^ =JCM 37538^T^), is an endophytic actinobacterium isolated from the gut contents of *Pheretima aspergillum* caught in the wild in Guangzhou, China. The GenBank/EMBL/DDBJ accession numbers for the 16S rRNA gene sequences of strains HD1123-B1^T^ is PP935229, respectively. The whole-genome shotgun project has been deposited at DDBJ/ENA/GenBank under the accession CP159919, respectively. The version described in this paper is the first version.

## SUPPLEMENTAL MATERIAL

Supplemental material spectrum.00717-25-s0001.docx Fig. S1 to S6; Tables S1 and S2.

ASM does not own the copyrights to Supplemental Material that may be linked to, or accessed through, an article. The authors have granted ASM a non-exclusive, world-wide license to publish the Supplemental Material files. Please contact the corresponding author directly for reuse.

## References

[1] Ma Y, Xu M, Liu H, Yu T, Guo P, Liu W, Jin X. 2021. Antimicrobial compounds were isolated from the secondary metabolites of Gordonia, a resident of intestinal tract of Periplaneta americana. AMB Express 11:111. doi:10.1186/s13568-021-01272-y 34331149 PMC8324697

[2] Law JW-F, Law LN-S, Letchumanan V, Tan LT-H, Wong SH, Chan K-G, Ab Mutalib N-S, Lee L-H. 2020. Anticancer drug discovery from microbial sources: the unique mangrove streptomycetes. Molecules 25:5365. doi:10.3390/molecules25225365 33212836 PMC7698459

[3] O’Shea AE, Valdera FA, Ensley D, Smolinsky TR, Cindass JL, Kemp Bohan PM, Hickerson AT, Carpenter EL, McCarthy PM, Adams AM, Vreeland TJ, Clifton GT, Peoples GE. 2022. Immunologic and dose dependent effects of rapamycin and its evolving role in chemoprevention. Clin Immunol 245:109095. doi:10.1016/j.clim.2022.109095 35973640

[4] Ehrenkaufer G, Li P, Stebbins EE, Kangussu-Marcolino MM, Debnath A, White CV, Moser MS, DeRisi J, Gisselberg J, Yeh E, Wang SC, Company AH, Monti L, Caffrey CR, Huston CD, Wang B, Singh U. 2020. Identification of anisomycin, prodigiosin and obatoclax as compounds with broad-spectrum anti-parasitic activity. PLoS Negl Trop Dis 14:e0008150. doi:10.1371/journal.pntd.0008150 32196500 PMC7112225

[5] Sánchez-Suárez J, Coy-Barrera E, Villamil L, Díaz L. 2020. Streptomyces-derived metabolites with potential photoprotective properties-A systematic literature review and meta-analysis on the reported chemodiversity. Molecules 25:3221. doi:10.3390/molecules25143221 32679651 PMC7397340

[6] Chater KF. 2006. Streptomyces inside-out: a new perspective on the bacteria that provide us with antibiotics . Phil Trans R Soc B 361:761–768. doi:10.1098/rstb.2005.1758 16627293 PMC1609407

[7] Chevrette MG, Currie CR. 2019. Emerging evolutionary paradigms in antibiotic discovery. J Ind Microbiol Biotechnol 46:257–271. doi:10.1007/s10295-018-2085-6 30269177

[8] Waksman SA, Henrici AT. 1943. The nomenclature and classification of the actinomycetes. J Bacteriol 46:337–341. doi:10.1128/jb.46.4.337-341.1943 16560709 PMC373826

[9] Arocha-Garza HF, Canales-Del Castillo R, Eguiarte LE, Souza V, De la Torre-Zavala S. 2017. High diversity and suggested endemicity of culturable actinobacteria in an extremely oligotrophic desert oasis. PeerJ 5:e3247. doi:10.7717/peerj.3247 28480140 PMC5417069

[10] van der Meij A, Worsley SF, Hutchings MI, van Wezel GP. 2017. Chemical ecology of antibiotic production by actinomycetes. FEMS Microbiol Rev 41:392–416. doi:10.1093/femsre/fux005 28521336

[11] Rice LB. 2008. Federal funding for the study of antimicrobial resistance in nosocomial pathogens: no ESKAPE. J Infect Dis 197:1079–1081. doi:10.1086/533452 18419525

[12] Kumar P, Bag S, Ghosh TS, Dey P, Dayal M, Saha B, Verma J, Pant A, Saxena S, Desigamani A, Rana P, Kumar D, Sharma NC, Hanpude P, Maiti TK, Mukhopadhyay AK, Bhadra RK, Nair GB, Ramamurthy T, Das B. 2017. Molecular insights into antimicrobial resistance traits of multidrug resistant enteric pathogens isolated from India. Sci Rep 7:14468. doi:10.1038/s41598-017-14791-1 29089611 PMC5663842

[13] Mancuso G, Midiri A, Gerace E, Biondo C. 2021. Bacterial antibiotic resistance: the most critical pathogens. Pathogens 10:1310. doi:10.3390/pathogens10101310 34684258 PMC8541462

[14] Lewis K. 2017. New approaches to antimicrobial discovery. Biochem Pharmacol 134:87–98. doi:10.1016/j.bcp.2016.11.002 27823963

[15] Wright GD. 2012. Antibiotics: a new hope. Chem Biol 19:3–10. doi:10.1016/j.chembiol.2011.10.019 22284349

[16] Van Arnam EB, Currie CR, Clardy J. 2018. Defense contracts: molecular protection in insect-microbe symbioses. Chem Soc Rev 47:1638–1651. doi:10.1039/c7cs00340d 28745342

[17] Sun J, Tian F, Zhang Y, Wu M, Mao R, Le Z, Xu D, Cao H, Ma Z. 2019. Chromatographic fingerprint and quantitative analysis of commercial Pheretima aspergillum (Guang Dilong) and its adulterants by UPLC-DAD. Int J Anal Chem 2019:4531092. doi:10.1155/2019/4531092 30728838 PMC6343145

[18] People’s Bank of China. 2010. State Pharmacopoeia Commission of the People’s Republic of China, “People’s Republic of China Financial Industry Standard, p 122. In In Chinese Law & Government

[19] Zhao J, Qi S-P, Wu J, Li L, He R-Q. 2005. Earthworm fibrinolytic enzyme. Studies in Natural Products Chemistry:825–847. doi:10.1016/S1572-5995(05)80048-1

[20] Sapkota R, Santos S, Farias P, Krogh PH, Winding A. 2020. Insights into the earthworm gut multi-kingdom microbial communities. Sci Total Environ 727:138301. doi:10.1016/j.scitotenv.2020.138301 32330704

[21] Yang Y, Callaham MA, Wu X, Zhang Y, Wu D, Wang D. 2023. Gut microbial communities and their potential roles in cellulose digestion and thermal adaptation of earthworms. Science of The Total Environment 903:166666. doi:10.1016/j.scitotenv.2023.166666 37657540

[22] Nwankwo IU, Edward KC, Udensi CG. 2023. Screening of bacteria isolates from earthworm cast for antibacterial activities. sa 22:83–94. doi:10.4314/sa.v22i2.9

[23] Balachandar R, Karmegam N, Saravanan M, Subbaiya R, Gurumoorthy P. 2018. Synthesis of bioactive compounds from vermicast isolated actinomycetes species and its antimicrobial activity against human pathogenic bacteria. Microb Pathog 121:155–165. doi:10.1016/j.micpath.2018.05.027 29778820

[24] Wu L, Bai S, Huang J, Cui G, Li Q, Wang J, Du X, Fu W, Li C, Wei W, Lin H, Luo M-L. 2023. Nigericin boosts anti-tumor immune response via inducing pyroptosis in triple-negative breast cancer. Cancers (Basel) 15:3221. doi:10.3390/cancers15123221 37370831 PMC10296105

[25] Steinrauf LK, Pinkerton M, Chamberlin JW. 1968. The structure of nigericin. Biochem Biophys Res Commun 33:29–31. doi:10.1016/0006-291x(68)90249-0 5696503

[26] Gao G, Liu F, Xu Z, Wan D, Han Y, Kuang Y, Wang Q, Zhi Q. 2021. Evidence of nigericin as a potential therapeutic candidate for cancers: a review. Biomedicine & Pharmacotherapy 137:111262. doi:10.1016/j.biopha.2021.111262 33508621

[27] Zhu X, Hong A, Sun X, Wang W, He G, Luo H, Wu Z, Xu Q, Hu Z, Wu X, Huang D, Li L, Zhao X, Deng X. 2022. Nigericin is effective against multidrug resistant gram-positive bacteria, persisters, and biofilms. Front Cell Infect Microbiol 12:1055929. doi:10.3389/fcimb.2022.1055929 36605124 PMC9807916

[28] Lin P-B, Shen J, Ou P-Y, Liu L-Y, Chen Z-Y, Chu F-J, Wang J, Jin X-B. 2019. Prodigiosin isolated from Serratia marcescens in the periplaneta americana gut and its apoptosis‑inducing activity in HeLa cells. Oncol Rep 41:3377–3385. doi:10.3892/or.2019.7089 30942457

[29] Atlas RM, Atlas RM. 2004. Handbook of Microbiological Media. CRC Press.

[30] Shirling EB, Gottlieb D. 1966. Methods for characterization of Streptomyces species. Int J Syst Bacteriol 16:313–340. doi:10.1099/00207713-16-3-313

[31] Waksman SA. 1967. The Actinomycetes. A summary of current knowledge

[32] Gerhardt P. 1994. Methods for general and molecular bacteriology. In Methods for General & Molecular Microbiology. Vol. 607.

[33] Sandoval-Powers M, Králová S, Nguyen G-S, Fawwal DV, Degnes K, Lewin AS, Klinkenberg G, Wentzel A, Liles MR. 2021. Streptomyces poriferorum sp. nov., a novel marine sponge-derived Actinobacteria species expressing anti-MRSA activity. Syst Appl Microbiol 44:126244. doi:10.1016/j.syapm.2021.126244 34392062

[34] Pridham TG, Gottlieb D. 1948. The utilization of carbon compounds by some Actinomycetales as an aid for species determination. J Bacteriol 56:107–114. doi:10.1128/jb.56.1.107-114.1948 16561537 PMC518551

[35] Christensen WB. 1946. Urea decomposition as a means of differentiating proteus and paracolon cultures from each other and from Salmonella and Shigella types. J Bacteriol 52:461–466. doi:10.1128/jb.52.4.461-466.1946 16561200 PMC518212

[36] Williams ST, Goodfellow M, Alderson G, Wellington EMH, Sneath PHA, Sackin MJ. 1983. Numerical classification of Streptomyces and related genera. J Gen Microbiol 129:1743–1813. doi:10.1099/00221287-129-6-1743 6631406

[37] Smibert R, Krieg N, Gerhardt P, Murray R, Wood W. 1994. Methods for General and Molecular Bacteriology.American Society for Microbiology, Washington, DC.

[38] Maiti PK, Mandal S. 2019. Majority of actinobacterial strains isolated from Kashmir Himalaya soil are rich source of antimicrobials and industrially important biomolecules. AiM 09:220–238. doi:10.4236/aim.2019.93016

[39] Cowan ST. 1974. Manual for the identification of medical bacteria

[40] Weisburg WG, Barns SM, Pelletier DA, Lane DJ. 1991. 16S ribosomal DNA amplification for phylogenetic study. J Bacteriol 173:697–703. doi:10.1128/jb.173.2.697-703.1991 1987160 PMC207061

[41] Kumar S, Stecher G, Li M, Knyaz C, Tamura K. 2018. MEGA X: molecular evolutionary genetics analysis across computing platforms. Mol Biol Evol 35:1547–1549. doi:10.1093/molbev/msy096 29722887 PMC5967553

[42] Kimura M. 1980. A simple method for estimating evolutionary rates of base substitutions through comparative studies of nucleotide sequences. J Mol Evol 16:111–120. doi:10.1007/BF01731581 7463489

[43] Trees RP. 1987. The neighbor-joining method: a new method for reconstructing phylogenetic trees. Mol Biol Evol 4:406–425. doi:10.1093/oxfordjournals.molbev.a040454 3447015

[44] Kluge AG, Farris JS. 1969. Quantitative phyletics and the evolution of anurans. Syst Biol 18:1–32. doi:10.1093/sysbio/18.1.1

[45] Felsenstein J. 1981. Evolutionary trees from DNA sequences: a maximum likelihood approach. J Mol Evol 17:368–376. doi:10.1007/BF01734359 7288891

[46] Felsenstein J. 1985. Confidence limits on phylogenies: an approach using the bootstrap. Evolution (N Y) 39:783–791. doi:10.1111/j.1558-5646.1985.tb00420.x 28561359

[47] Koren S, Walenz BP, Berlin K, Miller JR, Bergman NH, Phillippy AM. 2017. Canu: scalable and accurate long-read assembly via adaptive k -mer weighting and repeat separation . Genome Res 27:722–736. doi:10.1101/gr.215087.116 28298431 PMC5411767

[48] Hyatt D, Chen G-L, Locascio PF, Land ML, Larimer FW, Hauser LJ. 2010. Prodigal: prokaryotic gene recognition and translation initiation site identification. BMC Bioinformatics 11:119. doi:10.1186/1471-2105-11-119 20211023 PMC2848648

[49] Chan PP, Lowe TM, In. 2019. tRNAscan-SE: searching for tRNA genes in genomic sequences. Methods Mol Biol 1962:1–14. doi:10.1007/978-1-4939-9173-0_1 31020551 PMC6768409

[50] Nawrocki EP, Eddy SR. 2013. Infernal 1.1: 100-fold faster RNA homology searches. Bioinformatics 29:2933–2935. doi:10.1093/bioinformatics/btt509 24008419 PMC3810854

[51] Tarailo-Graovac M, Chen N. 2009. Using RepeatMasker to identify repetitive elements in genomic sequences. Curr Protoc Bioinformatics Chapter 4:4. doi:10.1002/0471250953.bi0410s25 19274634

[52] Bertelli C, Brinkman FSL. 2018. Improved genomic island predictions with IslandPath-DIMOB. Bioinformatics 34:2161–2167. doi:10.1093/bioinformatics/bty095 29905770 PMC6022643

[53] Akhter S, Aziz RK, Edwards RA. 2012. PhiSpy: a novel algorithm for finding prophages in bacterial genomes that combines similarity- and composition-based strategies. Nucleic Acids Res 40:e126–e126. doi:10.1093/nar/gks406 22584627 PMC3439882

[54] Blin K, Shaw S, Vader L, Szenei J, Reitz ZL, Augustijn HE, Cediel-Becerra JDD, de Crécy-Lagard V, Koetsier RA, Williams SE, et al.. 2025. antiSMASH 8.0: extended gene cluster detection capabilities and analyses of chemistry, enzymology, and regulation. Nucleic Acids Res 53:W32–W38. doi:10.1093/nar/gkaf334 40276974 PMC12230676

[55] Rangannan V, Bansal M. 2009. Relative stability of DNA as a generic criterion for promoter prediction: whole genome annotation of microbial genomes with varying nucleotide base composition. Mol Biosyst 5:1758–1769. doi:10.1039/B906535K 19593472

[56] Krzywinski M, Schein J, Birol I, Connors J, Gascoyne R, Horsman D, Jones SJ, Marra MA. 2009. Circos: an information aesthetic for comparative genomics. Genome Res 19:1639–1645. doi:10.1101/gr.092759.109 19541911 PMC2752132

[57] Aziz RK, Bartels D, Best AA, DeJongh M, Disz T, Edwards RA, Formsma K, Gerdes S, Glass EM, Kubal M, et al.. 2008. The RAST Server: rapid annotations using subsystems technology. BMC Genomics 9:75. doi:10.1186/1471-2164-9-75 18261238 PMC2265698

[58] Conesa A, Götz S, García-Gómez JM, Terol J, Talón M, Robles M. 2005. Blast2GO: a universal tool for annotation, visualization and analysis in functional genomics research. Bioinformatics 21:3674–3676. doi:10.1093/bioinformatics/bti610 16081474

[59] Lee I, Ouk Kim Y, Park S-C, Chun J. 2016. OrthoANI: an improved algorithm and software for calculating average nucleotide identity. Int J Syst Evol Microbiol 66:1100–1103. doi:10.1099/ijsem.0.000760 26585518

[60] Meier-Kolthoff JP, Klenk H-P, Göker M. 2014. Taxonomic use of DNA G+C content and DNA-DNA hybridization in the genomic age. Int J Syst Evol Microbiol 64:352–356. doi:10.1099/ijs.0.056994-0 24505073

[61] Meier-Kolthoff JP, Göker M. 2019. TYGS is an automated high-throughput platform for state-of-the-art genome-based taxonomy. Nat Commun 10:2182. doi:10.1038/s41467-019-10210-3 31097708 PMC6522516

[62] Ondov BD, Treangen TJ, Melsted P, Mallonee AB, Bergman NH, Koren S, Phillippy AM. 2016. Mash: fast genome and metagenome distance estimation using MinHash. Genome Biol 17:132. doi:10.1186/s13059-016-0997-x 27323842 PMC4915045

[63] Meier-Kolthoff JP, Auch AF, Klenk H-P, Göker M. 2013. Genome sequence-based species delimitation with confidence intervals and improved distance functions. BMC Bioinformatics 14:60. doi:10.1186/1471-2105-14-60 23432962 PMC3665452

[64] Lefort V, Desper R, Gascuel O. 2015. FastME 2.0: a comprehensive, accurate, and fast distance-based phylogeny inference program. Mol Biol Evol 32:2798–2800. doi:10.1093/molbev/msv150 26130081 PMC4576710

[65] Bhattacharjee A, Bayzid MS. 2020. Machine learning based imputation techniques for estimating phylogenetic trees from incomplete distance matrices. BMC Genomics 21:497. doi:10.1186/s12864-020-06892-5 32689946 PMC7370488

[66] Law JW-F, Tan K-X, Wong SH, Ab Mutalib N-S, Lee L-H. 2018. Taxonomic and Characterization Methods of Streptomyces: A Review. Prog Micobes Mol Biol 1. doi:10.36877/pmmb.a0000009

[67] Richter M, Rosselló-Móra R. 2009. Shifting the genomic gold standard for the prokaryotic species definition. Proc Natl Acad Sci USA 106:19126–19131. doi:10.1073/pnas.0906412106 19855009 PMC2776425

[68] Riesco R, Trujillo ME. 2024. Update on the proposed minimal standards for the use of genome data for the taxonomy of prokaryotes. Int J Syst Evol Microbiol 74:006300. doi:10.1099/ijsem.0.006300 38512750 PMC10963913

[69] Auch AF, von Jan M, Klenk H-P, Göker M. 2010. Digital DNA-DNA hybridization for microbial species delineation by means of genome-to-genome sequence comparison. Stand Genomic Sci 2:117–134. doi:10.4056/sigs.531120 21304684 PMC3035253

[70] Stackebrandt E, Goebel BM. 1994. Taxonomic note: a place for DNA-DNA reassociation and 16S rRNA sequence analysis in the present species definition in bacteriology. Int J Syst Evol Microbiol 44:846–849. doi:10.1099/00207713-44-4-846

[71] PRIDHAM TG, HESSELTINE CW, BENEDICT RG. 1958. A guide for the classification of streptomycetes according to selected groups; placement of strains in morphological sections. Appl Microbiol 6:52–79. doi:10.1128/am.6.1.52-79.1958 13509657 PMC1057356

[72] Nammali A, Intaraudom C, Pittayakhajonwut P, Suriyachadkun C, Tadtong S, Srabua P, Thawai C. 2021. Streptomyces coffeae sp. nov., an endophytic actinomycete isolated from the root of Coffea arabica (L.). Int J Syst Evol Microbiol 71. doi:10.1099/ijsem.0.004834 34106825

[73] Nammali A, Intaraudom C, Pittayakhajonwut P, Suriyachadkun C, Tadtong S, Tanasupawat S, Thawai C. 2021. Streptomyces endocoffeicus sp. nov., an endophytic actinomycete isolated from Coffea arabica (L.). Antonie Van Leeuwenhoek 114:1889–1898. doi:10.1007/s10482-021-01648-x 34480669

[74] Hamedi J, Mohammadipanah F, Klenk H-P, Pötter G, Schumann P, Spröer C, von Jan M, Kroppenstedt RM. 2010. Streptomyces iranensis sp. nov., isolated from soil. Int J Syst Evol Microbiol 60:1504–1509. doi:10.1099/ijs.0.015339-0 19684315

[75] Yang Z, Xie J, Fang J, Lv M, Yang M, Deng Z, Xie Y, Cai L. 2022. Nigericin exerts anticancer effects through inhibition of the SRC/STAT3/BCL-2 in osteosarcoma. Biochem Pharmacol 198:114938. doi:10.1016/j.bcp.2022.114938 35114189

[76] Dongiovanni P, Valenti L, Ludovica Fracanzani A, Gatti S, Cairo G, Fargion S. 2008. Iron depletion by deferoxamine up-regulates glucose uptake and insulin signaling in hepatoma cells and in rat liver. Am J Pathol 172:738–747. doi:10.2353/ajpath.2008.070097 18245813 PMC2258266

[77] Heskamp S, Raavé R, Boerman O, Rijpkema M, Goncalves V, Denat F. 2017. ^89^Zr-immuno-positron emission tomography in oncology: state-of-the-art ^89^Zr radiochemistry. Bioconjug Chem 28:2211–2223. doi:10.1021/acs.bioconjchem.7b00325 28767228 PMC5609224

[78] Klapschinski TA, Rabe P, Dickschat JS. 2016. Pristinol, a sesquiterpene alcohol with an unusual skeleton from Streptomyces pristinaespiralis. Angew Chem Int Ed Engl 55:10141–10144. doi:10.1002/anie.201605425 27403888

[79] Shen Q, Wang Q, Miao H, Shimada M, Utsumi M, Lei Z, Zhang Z, Nishimura O, Asada Y, Fujimoto N, Takanashi H, Akiba M, Shimizu K. 2022. Temperature affects growth, geosmin/2-methylisoborneol production, and gene expression in two cyanobacterial species. Environ Sci Pollut Res Int 29:12017–12026. doi:10.1007/s11356-021-16593-5 34558048

[80] Harvey BM, Mironenko T, Sun Y, Hong H, Deng Z, Leadlay PF, Weissman KJ, Haydock SF. 2007. Insights into polyether biosynthesis from analysis of the nigericin biosynthetic gene cluster in Streptomyces sp. DSM4137. Chem Biol 14:703–714. doi:10.1016/j.chembiol.2007.05.011 17584617

[81] Zhang L, Liu W, Xiao J, Hu T, Chen J, Chen K, Jiang H, Shen X. 2007. Malonyl-CoA: acyl carrier protein transacylase from Helicobacter pylori: Crystal structure and its interaction with acyl carrier protein. Protein Sci 16:1184–1192. doi:10.1110/ps.072757307 17525466 PMC2206670

[82] Keatinge-Clay AT, Shelat AA, Savage DF, Tsai SC, Miercke LJW, O’Connell JD 3rd, Khosla C, Stroud RM. 2003. Catalysis, specificity, and ACP docking site of Streptomyces coelicolor malonyl-CoA:ACP transacylase. Structure 11:147–154. doi:10.1016/s0969-2126(03)00004-2 12575934

[83] Chisuga T, Nagai A, Miyanaga A, Goto E, Kishikawa K, Kudo F, Eguchi T. 2022. Structural Insight into the Reaction Mechanism of Ketosynthase-Like Decarboxylase in a Loading Module of Modular Polyketide Synthases. ACS Chem Biol 17:198–206. doi:10.1021/acschembio.1c00856 34985877

[84] Sukjoi W, Ngamkham J, Attwood PV, Jitrapakdee S. 2021. Targeting Cancer Metabolism and Current Anti-Cancer Drugs. Adv Exp Med Biol 1286:15–48. doi:10.1007/978-3-030-55035-6_2 33725343

[85] Leulmi N, Sighel D, Defant A, Khenaka K, Boulahrouf A, Mancini I. 2019. Nigericin and grisorixin methyl ester from the Algerian soil-living Streptomyces youssoufiensis SF10 strain: a computational study on their epimeric structures and evaluation of glioblastoma stem cells growth inhibition. Nat Prod Res 33:266–273. doi:10.1080/14786419.2018.1446014 29513090

[86] Huang W, Briffotaux J, Wang X, Liu L, Hao P, Cimino M, Buchieri MV, Namouchi A, Ainsa J-A, Gicquel B. 2017. Ionophore A23187 shows anti-tuberculosis activity and synergy with tebipenem. Tuberculosis (Edinb) 107:111–118. doi:10.1016/j.tube.2017.09.001 29050757

[87] Gauba A, Rahman KM. 2023. Evaluation of antibiotic resistance mechanisms in gram-negative bacteria. Antibiotics (Basel) 12:1590. doi:10.3390/antibiotics12111590 37998792 PMC10668847

[88] Taechowisa T, Klomluam K, Chuen-Im T, S. Phutdha W. 2022. Synergistic antibacterial activity of 1-methyl ester-nigericin and methyl 5-(Hydroxymethyl) furan-2-carboxylate against proteus spp. Pakistan J of Biological Sciences 25:304–312. doi:10.3923/pjbs.2022.304.312 35638524

[89] Sahu AK, Said MS, Hingamire T, Gaur M, Khan A, Shanmugam D, Barvkar VT, Dharne MS, Bharde AA, Dastager SG. 2020. Approach to nigericin derivatives and their therapeutic potential. RSC Adv 10:43085–43091. doi:10.1039/d0ra05137c 35514935 PMC9058090

