# Supplementary material for: Streptomyces huangiella sp. nov., an endophytic actinomycete isolated from Pheretima aspergillum, a promising candidate for biological pathogen control
Source: Microbiol Spectr. 2025 Sep 17;13(10):e00717-25. doi: 10.1128/spectrum.00717-25 (PMC12502620; doi:10.1128/spectrum.00717-25)
Supplement: Supplemental material — Fig. S1 to S6; Tables S1 and S2. [file spectrum.00717-25-s0001.docx]

***Streptomyces huangiella*** **sp. nov., an endophytic actinomycete isolated from *Pheretima aspergillum* promising candidate for biological pathogen control**

Dan Huang**^1,2^**, Jinli Tan**^1,2^**, Jingyang Liao**^1,2^**, Liuchong Zhu**^1,2^**, Xiang Zhang**^1,2^**, Donghua Feng**^3^**, Wenbin Liu**^1,2*^**, Xiaobao Jin**^1,2*^**

*^1^School of basic medical sciences, Guangdong Pharmaceutical University, Guangzhou, PR China*

*^2^Guangdong Provincial Key Laboratory of Pharmaceutical Bioactive Substances, Guangdong Pharmaceutical University, Guangzhou, PR China*

*^3^Department of Laboratory Medicine, The First Affiliated Hospital of Guangzhou Medical University, Guangzhou Medical University, Guangzhou, China*

* **Correspondence**:

Corresponding Author **Xiaobao Jin**^∗^

[jinxf2001@163.com](mailto:jinxf2001@163.com)

Co-Corresponding **Author**^∗^

[408011126@gdpu.edu.cn](mailto:408011126@gdpu.edu.cn)

**Supplementary Figures and Tables**

**Figure S1.** Maximum-parsimony phylogenetic tree. Maximum-parsimony phylogenetic tree based on 16S rRNA gene sequences, showing the relationships between strains HD1123-B1^T^and related species of Genus *Streptomyces*. *Kitasatospora setae* KM-6054^T^ (NR_112082.2) was used as outgroups. Numbers at nodes refer to bootstrap values (based on 1000 replicates; only values > 50% were shown). Bar, 5.00 substitutions per nucleotide position.


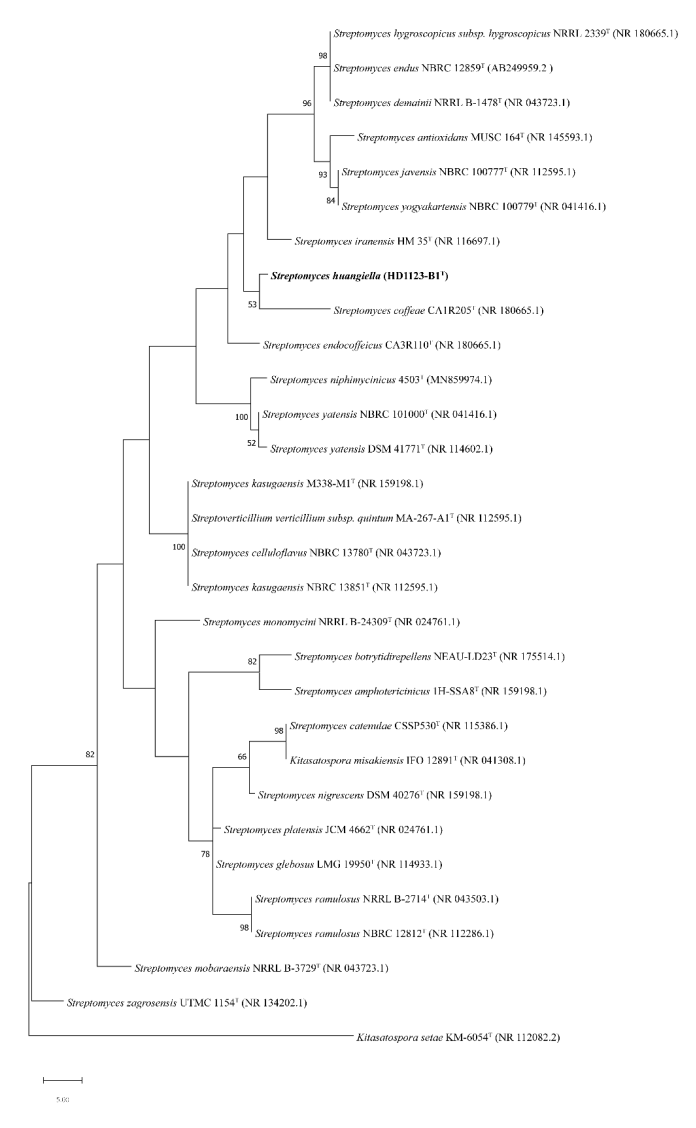


**Figure S2.** Maximum-likelihood phylogenetic tree. Maximum-likelihood phylogenetic tree based on 16S rRNA gene sequences, showing the relationships between strains HD1123-B1^T^and related species of Genus *Streptomyces*. *Kitasatospora setae* KM-6054^T^ (NR_112082.2) was used as outgroups. Numbers at nodes refer to bootstrap values (based on 1000 replicates; only values > 50% were shown). Bar, 0.01 substitutions per nucleotide position.


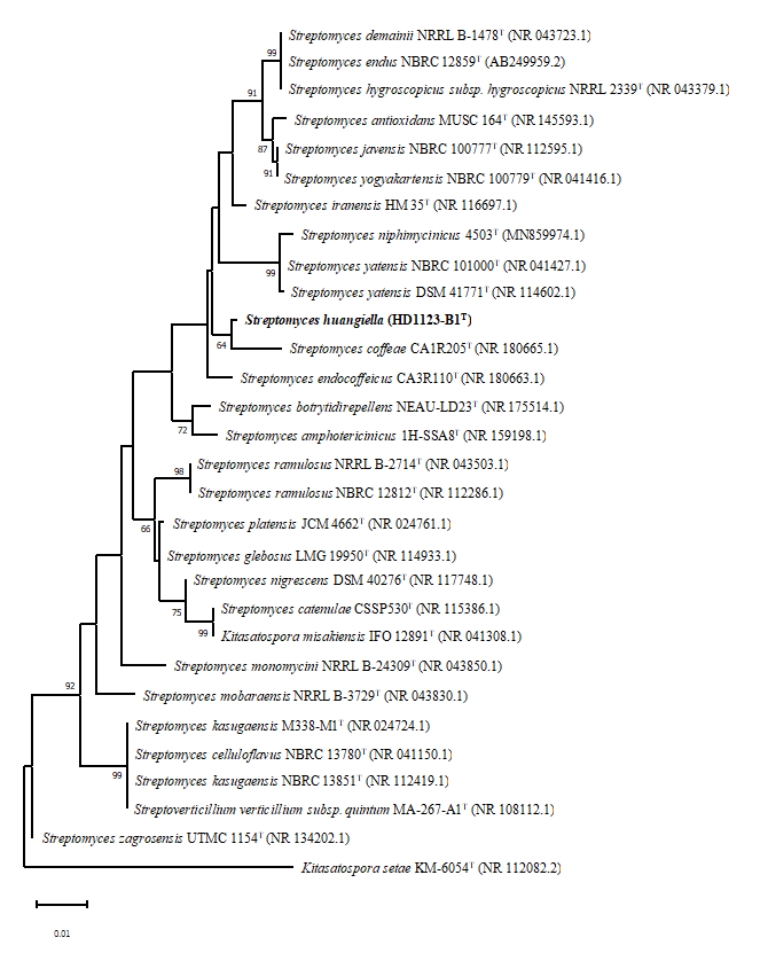


**Figure S3.** Taxonomic tools for the identification of novel *Streptomyces* species. Average nucleotide identity (ANI) heat map of ANI values between the closely related strains generated by OrthoANI software.


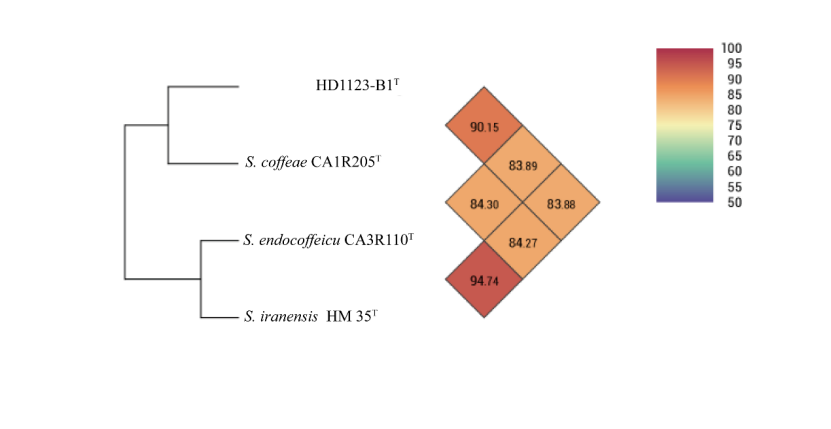


**Table S1.** Cultural characteristics on various media types of strains. All data were determined during this study.

| Medium | *Streptomyces huangiella*  sp.nov | *Streptomyces coffeae* CA1R205^T^ | *Streptomyces endocoffeicus* CA3R110^T^ | *Streptomyces iranensis* HM 35^T^ |
| --- | --- | --- | --- | --- |
| ISP 1 |  |  |  |  |
| Growth | Moderate | N | N | N |
| Aerial mycelium | white | N | N | N |
| Substrate mycelium | Grayish brown | N | N | N |
| Pigment | - | N | N | N |
| ISP 2 |  |  |  |  |
| Growth | Good | Good | Good | Good |
| Aerial mycelium | white | - | Greyish-white | Greyish-white to Grey |
| Substrate mycelium | Brownish black | Dark brownish  black | Pale greenish  yellow | Beige |
| Pigment | Dark grayish brown | - | - | Pale yellow |
| ISP 3 |  |  |  |  |
| Growth | Good | Good | Moderate | Good |
| Aerial mycelium | white | Greyish-white | Greyish-white | Greyish-white to Grey |
| Substrate mycelium | Brownish black | Dark brownish  black | Pale greenish  yellow | Brownish-yellow |
| Pigment | - | Dark yellowish brown | - | - |
| ISP 4 |  |  |  |  |
| Growth | Good | Good | Good | Moderate |
| Aerial mycelium | white | Greyish-white | Greyish-white | Grey |
| Substrate mycelium | Brownish black | Dark brownish  black | Pale greenish yellow | Light brownish-yellow |
| Pigment | - | Dark yellowish brown | - | - |
| ISP 5 |  |  |  |  |
| Growth | Moderate | Good | Good | Moderate |
| Aerial mycelium | - | Greyish-white | Greyish-white | Greyish-white |
| Substrate mycelium | Brownish black | Dark brownish  black | Strong greenish yellow | Light brownish-yellow |
| Pigment | Strong yellow green | Light yellowish-brown | Brilliant greenish yellow | - |
| ISP 6 |  |  |  |  |
| Growth | Poor | Moderate | Good | Poor |
| Aerial mycelium | - | - | Greyish-white | - |
| Substrate mycelium | Grayish yellow | Light yellowishbrown | Pale greenish yellow | Light yellowish-brown |
| Pigment | - | Light yellowish-brown | - | - |
| Gause’s Synthetic Agar |  |  |  |  |
| Growth | Good | N | N | N |
| Aerial mycelium | white | N | N | N |
| Substrate mycelium | Brownish black | N | N | N |
| Pigment | - | N | N | N |
| Czapek's Dox Agar |  |  |  |  |
| Growth | Good | Poor | Good | Poor |
| Aerial mycelium | White | Greyish-white | Greyish-white | Greyish-white |
| Substrate mycelium | Brownish gray | Dark brownish  black | Yellowish white | Yellowish-white |
| Pigment | - | - | - | - |
| Nutrient Agar |  |  |  |  |
| Growth | Good | Moderate | Moderate | Moderate |
| Aerial mycelium | - | Greyish-white | Greyish-white | Greyish-white |
| Substrate mycelium | Yellowish white | Yellowish-brown | Grayish greenish yellow | Light brownish-yellow |
| Pigment | - | - | - | - |
| Luria-Bertani Agar |  |  |  |  |
| Growth | Good | N | N | N |
| Aerial mycelium | - | N | N | N |
| Substrate mycelium | Brownish black | N | N | N |
| Pigment | - | N | N | N |
| Soybean Casein Digest Agar |  |  |  |  |
| Growth | Moderate | N | N | N |
| Aerial mycelium | - | N | N | N |
| Substrate mycelium | Pale yellow | N | N | N |
| Pigment | - | N | N | N |

+, positive; –, negative; (w), weekly positive; N, no date

**Figure S4.** Gram stain diagram of strain HD1123-B1^T^. The strain was Gram- positive

and showed filamentous structures when viewed under a light microscope (100×10).


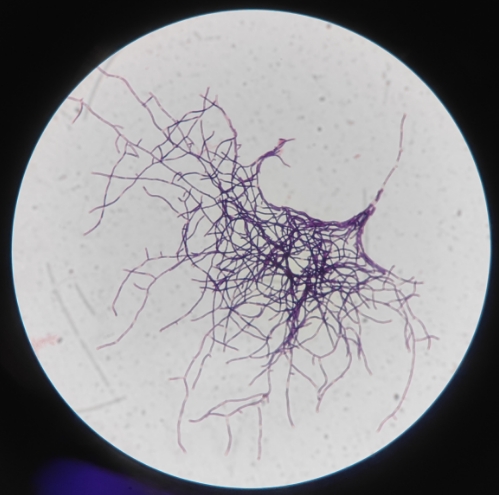


**Figure S5.** Scanning electron micrograph (SEM) of strain HD1123-B1^T^ showing abundant aerial hyphae following growth on ISP 2 at 28°C for 14 days. Bar:5.0,10.0 µm.


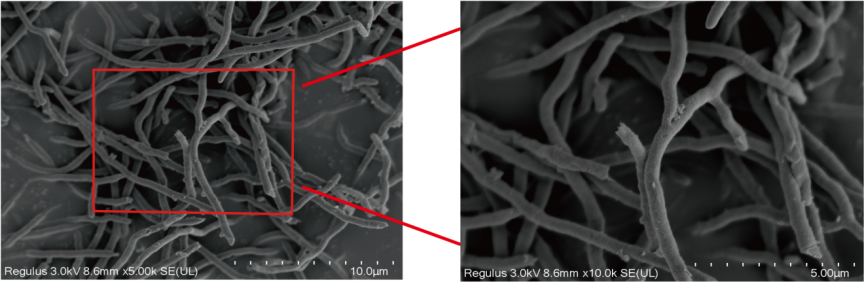


**Figure S6.** Genome annotations of strain HD1123-B1^T^. GO function annotates categorical statistics(a), eggNOG functional classification statistical map of functional genes(b) and KEGG annotates categorical statistics(c).


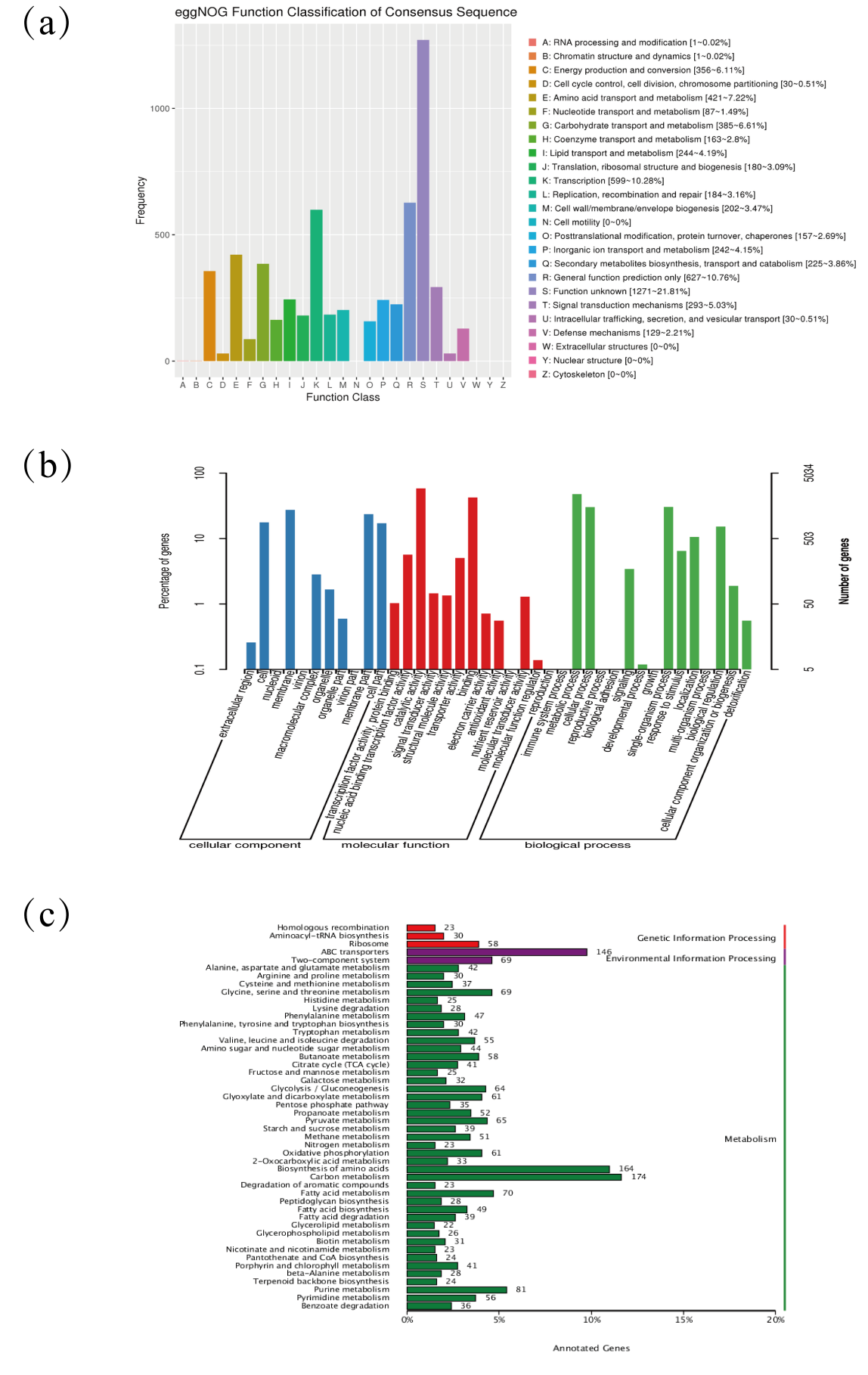


**Table S2.** The inhibitory zone size of the crude extract of the strain HD1123-B1^T^ against pathogenic bacteria (mm).

| **Bioassay microorganism** | **Strain HD1123-B1^T^** |
| --- | --- |
|  | **Distance of inhibition (mm)** |
| **Gram-positive bacteria** |  |
| *Staphylococcus aureus* ATCC 25923 | 23.80 |
| Methicillin-resistant *Staphylococcus aureus* (MRSA) ATCC 25213 | 24.18 |
| *Bacillus subtilis* ATCC 6633 | 26.68 |
| *Enterococcus faecalis* ATCC 51299 | 26.30 |
| **Gram-negative bacteria** |  |
| *Klebsiella pneumoniae* ATCC 13883 | - |
| *Escherichia coli* ATCC 25922 | - |
| *Pseudomonas aeruginosa* ATCC 25924 | - |
| *Ralstonia solanacearum* GIM 1.70 | 24.80 |
| **Fungi** |  |
| *Candida albicans* ATCC 10231 | - |

–, negative
